# Using deep learning to probe the neural code for images in primary visual cortex

**DOI:** 10.1167/19.4.29

**Published:** 2019-04-26

**Authors:** William F. Kindel, Elijah D. Christensen, Joel Zylberberg

**Affiliations:** william.kindel@ucdenver.edu; Department of Physiology and Biophysics, University of Colorado School of Medicine, Aurora, CO, USA; Department of Physiology and Biophysics, University of Colorado School of Medicine, Aurora, CO, USA; Department of Physiology and Biophysics, University of Colorado School of Medicine, Aurora, CO, USA; Learning in Machines and Brains Program, Canadian Institute for Advanced Research, Toronto, Canada

**Keywords:** *neural coding*, *artificial neural networks*, *primary visual cortex*, *receptive fields*

## Abstract

Primary visual cortex (V1) is the first stage of cortical image processing, and major effort in systems neuroscience is devoted to understanding how it encodes information about visual stimuli. Within V1, many neurons respond selectively to edges of a given preferred orientation: These are known as either *simple* or *complex cells*. Other neurons respond to localized center–surround image features. Still others respond selectively to certain image stimuli, but the specific features that excite them are unknown. Moreover, even for the simple and complex cells—the best-understood V1 neurons—it is challenging to predict how they will respond to natural image stimuli. Thus, there are important gaps in our understanding of how V1 encodes images. To fill this gap, we trained deep convolutional neural networks to predict the firing rates of V1 neurons in response to natural image stimuli, and we find that the predicted firing rates are highly correlated ($\newcommand{\bialpha}{\boldsymbol{\alpha}}\newcommand{\bibeta}{\boldsymbol{\beta}}\newcommand{\bigamma}{\boldsymbol{\gamma}}\newcommand{\bidelta}{\boldsymbol{\delta}}\newcommand{\bivarepsilon}{\boldsymbol{\varepsilon}}\newcommand{\bizeta}{\boldsymbol{\zeta}}\newcommand{\bieta}{\boldsymbol{\eta}}\newcommand{\bitheta}{\boldsymbol{\theta}}\newcommand{\biiota}{\boldsymbol{\iota}}\newcommand{\bikappa}{\boldsymbol{\kappa}}\newcommand{\bilambda}{\boldsymbol{\lambda}}\newcommand{\bimu}{\boldsymbol{\mu}}\newcommand{\binu}{\boldsymbol{\nu}}\newcommand{\bixi}{\boldsymbol{\xi}}\newcommand{\biomicron}{\boldsymbol{\micron}}\newcommand{\bipi}{\boldsymbol{\pi}}\newcommand{\birho}{\boldsymbol{\rho}}\newcommand{\bisigma}{\boldsymbol{\sigma}}\newcommand{\bitau}{\boldsymbol{\tau}}\newcommand{\biupsilon}{\boldsymbol{\upsilon}}\newcommand{\biphi}{\boldsymbol{\phi}}\newcommand{\bichi}{\boldsymbol{\chi}}\newcommand{\bipsi}{\boldsymbol{\psi}}\newcommand{\biomega}{\boldsymbol{\omega}}{\overline {{\bf{CC}}} _{{\bf{norm}}}}$ = 0.556 ± 0.01) with the neurons' actual firing rates over a population of 355 neurons. This performance value is quoted for all neurons, with no selection filter. Performance is better for more active neurons: When evaluated only on neurons with mean firing rates above 5 Hz, our predictors achieve correlations of $\newcommand{\bialpha}{\boldsymbol{\alpha}}\newcommand{\bibeta}{\boldsymbol{\beta}}\newcommand{\bigamma}{\boldsymbol{\gamma}}\newcommand{\bidelta}{\boldsymbol{\delta}}\newcommand{\bivarepsilon}{\boldsymbol{\varepsilon}}\newcommand{\bizeta}{\boldsymbol{\zeta}}\newcommand{\bieta}{\boldsymbol{\eta}}\newcommand{\bitheta}{\boldsymbol{\theta}}\newcommand{\biiota}{\boldsymbol{\iota}}\newcommand{\bikappa}{\boldsymbol{\kappa}}\newcommand{\bilambda}{\boldsymbol{\lambda}}\newcommand{\bimu}{\boldsymbol{\mu}}\newcommand{\binu}{\boldsymbol{\nu}}\newcommand{\bixi}{\boldsymbol{\xi}}\newcommand{\biomicron}{\boldsymbol{\micron}}\newcommand{\bipi}{\boldsymbol{\pi}}\newcommand{\birho}{\boldsymbol{\rho}}\newcommand{\bisigma}{\boldsymbol{\sigma}}\newcommand{\bitau}{\boldsymbol{\tau}}\newcommand{\biupsilon}{\boldsymbol{\upsilon}}\newcommand{\biphi}{\boldsymbol{\phi}}\newcommand{\bichi}{\boldsymbol{\chi}}\newcommand{\bipsi}{\boldsymbol{\psi}}\newcommand{\biomega}{\boldsymbol{\omega}}{\overline {{\bf{CC}}} _{{\bf{norm}}}}$ = 0.69 ± 0.01 with the neurons' true firing rates. We find that the firing rates of both orientation-selective and non-orientation-selective neurons can be predicted with high accuracy. Additionally, we use a variety of models to benchmark performance and find that our convolutional neural-network model makes more accurate predictions.

## Introduction

Our ability to see arises because of the activity evoked in our brains as we view the world around us. Ever since Hubel and Wiesel (1959) mapped the flow of visual information from the retina to thalamus and then cortex, understanding how these different regions encode and process visual information has been a major focus of visual systems neuroscience. In the first cortical layer of visual processing—primary visual cortex (V1)—Hubel and Wiesel identified neurons that respond to oriented edges within image stimuli. These are called *simple* or *complex cells*, depending on how sensitive their responses are to shifts in the position of the edge. The simple and complex cells are well studied (Lehky, Sejnowski, & Desimone, 1992; David, Vinje, & Gallant, 2004; Montijn, Meijer, Lansink, & Pennartz, 2016). However, many V1 neurons are neither simple nor complex cells, and the classical models of simple and complex cells often fail to predict how those neurons will respond to naturalistic stimuli (Olshausen & Field, 2005). Thus, much of how V1 encodes visual information remains unknown. We use deep learning to address this longstanding problem.

Recent advances in neural-recording technology and machine learning have put solving the V1 neural code within reach. Experimental technology for simultaneously recording from large populations of neurons—such as multielectrode arrays—has opened the door to studying how the collective behavior of neurons encodes sensory information. Moreover, methods of machine learning inspired by the anatomy of the mammalian visual system, known as *convolutional neural networks*, have achieved impressive success in increasingly difficult image-classification tasks (Krizhevsky, Sutskever, & Hinton, 2012; LeCun, Bengio, & Hinton, 2015). Recently, these artificial neural networks have been used to study the visual system (Yamins & DiCarlo, 2016), setting the state of the art for predicting stimulus-evoked neural activity in the retina (McIntosh, Maheswaranathan, Nayebi, Ganguli, & Baccus, 2016) and inferior temporal cortex (Yamins et al., 2014). Despite these successes, we have not yet achieved a full understanding of how V1 represents natural images.

In this work, we present a convolutional neural network that predicts V1 activity patterns evoked by natural image stimuli. We use this network to predict the activity of 355 individual neurons in macaque-monkey V1, in which it represents the neural visual code for many neurons regardless of cell type. On held-out validation data, the network predicts firing rates that are highly correlated ($\newcommand{\bialpha}{\boldsymbol{\alpha}}\newcommand{\bibeta}{\boldsymbol{\beta}}\newcommand{\bigamma}{\boldsymbol{\gamma}}\newcommand{\bidelta}{\boldsymbol{\delta}}\newcommand{\bivarepsilon}{\boldsymbol{\varepsilon}}\newcommand{\bizeta}{\boldsymbol{\zeta}}\newcommand{\bieta}{\boldsymbol{\eta}}\newcommand{\bitheta}{\boldsymbol{\theta}}\newcommand{\biiota}{\boldsymbol{\iota}}\newcommand{\bikappa}{\boldsymbol{\kappa}}\newcommand{\bilambda}{\boldsymbol{\lambda}}\newcommand{\bimu}{\boldsymbol{\mu}}\newcommand{\binu}{\boldsymbol{\nu}}\newcommand{\bixi}{\boldsymbol{\xi}}\newcommand{\biomicron}{\boldsymbol{\micron}}\newcommand{\bipi}{\boldsymbol{\pi}}\newcommand{\birho}{\boldsymbol{\rho}}\newcommand{\bisigma}{\boldsymbol{\sigma}}\newcommand{\bitau}{\boldsymbol{\tau}}\newcommand{\biupsilon}{\boldsymbol{\upsilon}}\newcommand{\biphi}{\boldsymbol{\phi}}\newcommand{\bichi}{\boldsymbol{\chi}}\newcommand{\bipsi}{\boldsymbol{\psi}}\newcommand{\biomega}{\boldsymbol{\omega}}{\overline {{\rm{CC}}} _{{\rm{norm}}}} = {0}.{556}\ \pm\ {0}.{015}$) with the neurons' actual firing rates. This performance value is quoted for all neurons, with no selection filter. Performance is better for more active neurons: When evaluated only on neurons with mean firing rates above 5 Hz, our predictors achieve correlations of $\newcommand{\bialpha}{\boldsymbol{\alpha}}\newcommand{\bibeta}{\boldsymbol{\beta}}\newcommand{\bigamma}{\boldsymbol{\gamma}}\newcommand{\bidelta}{\boldsymbol{\delta}}\newcommand{\bivarepsilon}{\boldsymbol{\varepsilon}}\newcommand{\bizeta}{\boldsymbol{\zeta}}\newcommand{\bieta}{\boldsymbol{\eta}}\newcommand{\bitheta}{\boldsymbol{\theta}}\newcommand{\biiota}{\boldsymbol{\iota}}\newcommand{\bikappa}{\boldsymbol{\kappa}}\newcommand{\bilambda}{\boldsymbol{\lambda}}\newcommand{\bimu}{\boldsymbol{\mu}}\newcommand{\binu}{\boldsymbol{\nu}}\newcommand{\bixi}{\boldsymbol{\xi}}\newcommand{\biomicron}{\boldsymbol{\micron}}\newcommand{\bipi}{\boldsymbol{\pi}}\newcommand{\birho}{\boldsymbol{\rho}}\newcommand{\bisigma}{\boldsymbol{\sigma}}\newcommand{\bitau}{\boldsymbol{\tau}}\newcommand{\biupsilon}{\boldsymbol{\upsilon}}\newcommand{\biphi}{\boldsymbol{\phi}}\newcommand{\bichi}{\boldsymbol{\chi}}\newcommand{\bipsi}{\boldsymbol{\psi}}\newcommand{\biomega}{\boldsymbol{\omega}}{\overline {{\rm{CC}}} _{{\rm{norm}}}} = {0}.{69}\ \pm\ {0}.{01}$ with the neurons' true firing rates. Our deep network is overall more accurate than a library of other models used as a baseline for comparison.

## Methods

### Experimental data

**Figure 1:**
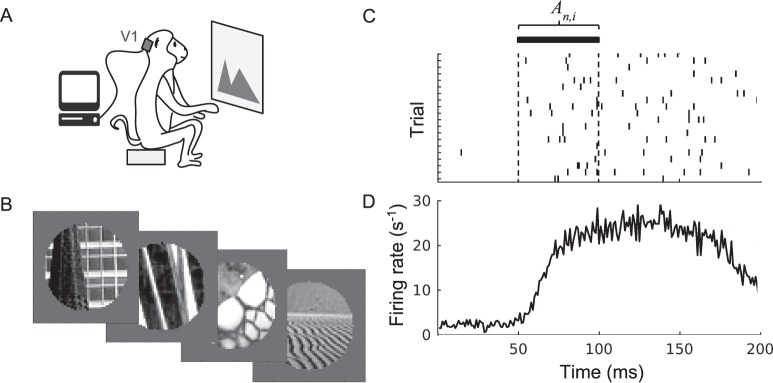
Experimental data collection and processing. (A) Neural activity was recorded in monkeys' V1 as they were shown a series of images. (B) The image set contains 270 circularly cropped natural images. (C) The response of a single neuron over repeated presentations of an image. Ticks indicate the neuron's spiking; each row corresponds to a different image-presentation trial. During the response window, the firing rate is computed and then averaged over trials to yield the average response A_n__,__i_ used in our analysis. (D) The neuron responds to image stimuli with a latency of ∼50 ms from the image onset at t = 0, as seen in the peristimulus time histogram (firing rate plotted against time, averaged over all 270 images).

We used publicly available multielectrode recordings from macaque V1 downloaded from the Collaborative Research in Computational Neuroscience website (http://crcns.org; Coen-Cagli, Kohn, & Schwartz, 2015). In these experiments, macaque monkeys were anesthetized and then presented with a series of images while the experimenters recorded the spiking activity of a population of neurons in V1 (Figure 1A and 1B) with a multielectrode array. Each image was presented for 100 ms, and there was a 200-ms blank screen shown between images. These recordings were conducted in 10 experimental sessions with three different animals, resulting in recordings from a total of 392 spike-sorted neurons whose receptive fields were centered on the stimulus. In the publicly available data, both well-isolated single units and small multiunit clusters are present. In our main analysis, we consider all of these as neurons; we also separately performed an analysis in which we attempted to distinguish between the single neurons and the small multiunit clusters. That result is included in the Discussion. A full description of the data and experimental methods is given by Coen-Cagli et al. (2015). Unlike those researchers, who used selection criteria based on responses to visual stimuli and reported results from a subset of 207 neurons, we used no further selection criteria and used all 392 spike-sorted and centered neurons.

We used 37 of these neurons from one experimental session to determine how to construct our network (its hyperparameters), and the remaining 355 neurons to evaluate its performance. For each neuron *n*, we calculated the mean firing rate *A_n_*_,_*_i_* evoked by each image *i* by averaging its firing rate across the 20 repeated presentations of that image. The firing rates were calculated over a window from 50 to 100 ms after the image was presented, to account for the signal-propagation delay from retina to V1 (Figure 1D; V1 firing rates increase dramatically at ∼50 ms after stimulus onset). We separately analyzed firing rates computed over a longer (100-ms) window, from 50 to 150 ms after stimulus onset; the results of that analysis are presented in the Discussion section.

We analyzed the responses to 270 natural images circularly cropped with a 1° aperture (Figure 1B). All 392 neurons are centered such that the 1° image aperture fully contains every neuron's receptive field. The full data set contains responses to natural and artificial stimuli, both full-size and cropped. We used only natural images because we are interested in the real-world behavior of the visual system, and we used only the cropped images because they have the same visual field as the grating stimuli that we used to characterize the neurons as either orientation selective or not.

### Deep neural-network model

**Figure 2:**
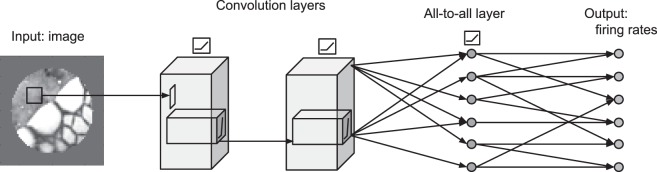
The optimized architecture of the deep convolutional-neural-network model. The network's inputs are the pixel values of an image, and each output unit gives the predicted firing rate of a single neuron in monkey V1.

To construct our predictive network, we used a convolutional neural network (CNN) whose input is an image and whose output is the predicted firing rates of every neuron in a given experimental session. Prior to training the neural network, we down-sampled the images using a nonoverlapping 2 × 2 window and cropped them to a size of 33 × 33 pixels. As shown in Figure 2, the network consists of a series of linear–nonlinear layers. The first layer(s) performs local feature extraction on the image by sweeping banks of convolutional filters over the image and then applying a maximum pooling operation. These local features are then globally combined at the all-to-all layer(s) to generate the predicted firing rate for every neuron in that data session.^1^

**Figure 3:**
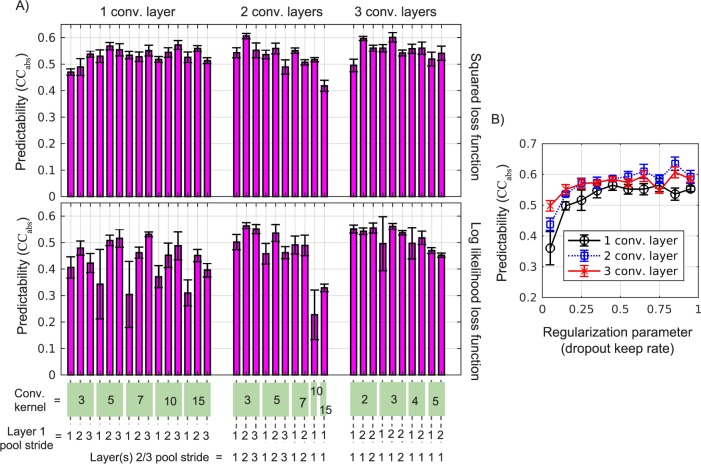
The hyperparameter optimization of the deep convolutional-neural-network model. (A) Adjusting the number of convolutional layers, loss function, convolutional kernel size (size of filters), and maxpool strides (scale of down-sampling) for just Layer 1 and both Layers 2 and 3. Each point is computed from the average Pearson correlation coefficient between the model's predictions and measured firing rates on one of the 10 experimental sessions with the standard error computed from five distinct partitions of training and evaluation data. (B) Adjusting the dropout keep rate for the best-performing networks with one, two, and three convolutional layers.

The number of each type of layer (convolutional with maximum pooling or all-to-all) and the details about each layer (number of units, convolution stride, etc.) were optimized to maximize the accuracy of the neural-activity predictions on the 37 neurons recorded in the second experimental session. We did this using a combination of manual and automated searches, where the results of our manual search informed the range of the hyperparameter space for an automated random search (Bergstra & Bengio, 2012). A subset of the results from the manual search is shown in Figure 3A and 3B. In Figure 3A, the number of convolutional layers, the kernel size of the convolutions, the pooling stride, and the loss function are adjusted. During training, units are randomly silenced (dropped out), which is a commonly used method for preventing overfitting in neural networks (Srivastava, Hinton, Krizhevsky, Sutskever, & Salakhutdinov, 2014). In Figure 3B, we take the best-performing networks with one, two, and three convolutional layers and adjust the dropout keep rate. Using the best-performing set of parameters, we defined our best CNN, denoted CNN2 because it is a two-convolutional-layer network. We trained and evaluated CNN2 using the data from the remaining nine experimental sessions.

For each experimental session, we trained our network using a cross-validation procedure where we randomly subdivided the given data set into a training subset (80% of the images and corresponding V1 activity patterns) and an evaluation subset (20% of the images). We then trained all layers of our network using the TensorFlow Python package with the gradient-descent optimizer. Based on the results of our hyperparameter search, which showed that this loss function outperforms the alternative log-likelihood one, we attributed a loss

\newcommand{\bialpha}{\boldsymbol{\alpha}}\newcommand{\bibeta}{\boldsymbol{\beta}}\newcommand{\bigamma}{\boldsymbol{\gamma}}\newcommand{\bidelta}{\boldsymbol{\delta}}\newcommand{\bivarepsilon}{\boldsymbol{\varepsilon}}\newcommand{\bizeta}{\boldsymbol{\zeta}}\newcommand{\bieta}{\boldsymbol{\eta}}\newcommand{\bitheta}{\boldsymbol{\theta}}\newcommand{\biiota}{\boldsymbol{\iota}}\newcommand{\bikappa}{\boldsymbol{\kappa}}\newcommand{\bilambda}{\boldsymbol{\lambda}}\newcommand{\bimu}{\boldsymbol{\mu}}\newcommand{\binu}{\boldsymbol{\nu}}\newcommand{\bixi}{\boldsymbol{\xi}}\newcommand{\biomicron}{\boldsymbol{\micron}}\newcommand{\bipi}{\boldsymbol{\pi}}\newcommand{\birho}{\boldsymbol{\rho}}\newcommand{\bisigma}{\boldsymbol{\sigma}}\newcommand{\bitau}{\boldsymbol{\tau}}\newcommand{\biupsilon}{\boldsymbol{\upsilon}}\newcommand{\biphi}{\boldsymbol{\phi}}\newcommand{\bichi}{\boldsymbol{\chi}}\newcommand{\bipsi}{\boldsymbol{\psi}}\newcommand{\biomega}{\boldsymbol{\omega}}\begin{equation}\tag{1}{L_n} = {{\sum\nolimits_i {({y_{n,i}}} - {A_{n,i}}{)^2}} \over {{\rm{va}}{{\rm{r}}_i}({A_{n,i}})}}\end{equation}

to each neuron (indexed by *n*), where *i* is the image index, *A_n_*_,_*_i_* the measured response, and *y* the network's predicted response. The neurons' losses are summed, yielding the total loss used by the optimizer. To ensure that the performance generalizes, the training data were subdivided into data used by the optimizer to train the weights (66% of the images) and another small subset (14% of the images) to stop the training when accuracy stops improving (early stopping).

To quantify the performance of the predictor, we compared the network's predicted firing rates to the neurons' measured firing rates using a held-out evaluation set. This set was used neither to determine the hyperparameters nor to train the weights in our neural network. We calculated the Pearson correlation coefficient $\newcommand{\bialpha}{\boldsymbol{\alpha}}\newcommand{\bibeta}{\boldsymbol{\beta}}\newcommand{\bigamma}{\boldsymbol{\gamma}}\newcommand{\bidelta}{\boldsymbol{\delta}}\newcommand{\bivarepsilon}{\boldsymbol{\varepsilon}}\newcommand{\bizeta}{\boldsymbol{\zeta}}\newcommand{\bieta}{\boldsymbol{\eta}}\newcommand{\bitheta}{\boldsymbol{\theta}}\newcommand{\biiota}{\boldsymbol{\iota}}\newcommand{\bikappa}{\boldsymbol{\kappa}}\newcommand{\bilambda}{\boldsymbol{\lambda}}\newcommand{\bimu}{\boldsymbol{\mu}}\newcommand{\binu}{\boldsymbol{\nu}}\newcommand{\bixi}{\boldsymbol{\xi}}\newcommand{\biomicron}{\boldsymbol{\micron}}\newcommand{\bipi}{\boldsymbol{\pi}}\newcommand{\birho}{\boldsymbol{\rho}}\newcommand{\bisigma}{\boldsymbol{\sigma}}\newcommand{\bitau}{\boldsymbol{\tau}}\newcommand{\biupsilon}{\boldsymbol{\upsilon}}\newcommand{\biphi}{\boldsymbol{\phi}}\newcommand{\bichi}{\boldsymbol{\chi}}\newcommand{\bipsi}{\boldsymbol{\psi}}\newcommand{\biomega}{\boldsymbol{\omega}}{\rm{CC}}_{{\rm{abs}}}^{{\rm{CNN2}}}$ between the predicted and measured firing rates for each neuron. Following the convention of Schoppe, Harper, Willmore, King, and Schnupp (2016), we scaled the Pearson correlation coefficient by its theoretical maximum value given neural variability to yield the normalized Pearson correlation coefficient

\newcommand{\bialpha}{\boldsymbol{\alpha}}\newcommand{\bibeta}{\boldsymbol{\beta}}\newcommand{\bigamma}{\boldsymbol{\gamma}}\newcommand{\bidelta}{\boldsymbol{\delta}}\newcommand{\bivarepsilon}{\boldsymbol{\varepsilon}}\newcommand{\bizeta}{\boldsymbol{\zeta}}\newcommand{\bieta}{\boldsymbol{\eta}}\newcommand{\bitheta}{\boldsymbol{\theta}}\newcommand{\biiota}{\boldsymbol{\iota}}\newcommand{\bikappa}{\boldsymbol{\kappa}}\newcommand{\bilambda}{\boldsymbol{\lambda}}\newcommand{\bimu}{\boldsymbol{\mu}}\newcommand{\binu}{\boldsymbol{\nu}}\newcommand{\bixi}{\boldsymbol{\xi}}\newcommand{\biomicron}{\boldsymbol{\micron}}\newcommand{\bipi}{\boldsymbol{\pi}}\newcommand{\birho}{\boldsymbol{\rho}}\newcommand{\bisigma}{\boldsymbol{\sigma}}\newcommand{\bitau}{\boldsymbol{\tau}}\newcommand{\biupsilon}{\boldsymbol{\upsilon}}\newcommand{\biphi}{\boldsymbol{\phi}}\newcommand{\bichi}{\boldsymbol{\chi}}\newcommand{\bipsi}{\boldsymbol{\psi}}\newcommand{\biomega}{\boldsymbol{\omega}}\begin{equation}\tag{2}{\rm{CC}}_{{\rm{norm}}}^{{\rm{CNN2}}}{\rm{ = }}{{{\rm{CC}}_{{\rm{abs}}}^{{\rm{CNN2}}}} \over {{\rm{C}}{{\rm{C}}_{\max }}}}\end{equation}

that we use to quantify our results. Thus in principle, a perfect model can achieve CC_norm_ = 1.

To compute CC_max_, we followed a bootstrapping procedure (in contrast to Schoppe et al., 2016) where we generated fake data by drawing random numbers from Gaussian distributions with the same statistics as the measured neural data. For each neuron and image, we averaged over 20 of these values to obtain a simulated prediction. We then computed the correlation between these simulated predictions and the neurons' actual mean firing rates to find the maximum correlation CC_max_ possible given the variability in stimulus-evoked neural firing rates. While we acknowledge that neural firing rates are not Gaussian distributed, the CC_max_ estimate, being a second-order statistic of the neural firing rates (and their estimates via the predictor networks), is sensitive only to the first- and second-order statistics of the neural data. A Gaussian distribution captures these first- and second-order statistics while making as few assumptions as possible about the higher order statistics in the data (i.e., it is a second-order *maximum entropy* model). As a result, our use of Gaussian distributions does not affect the reliability of our estimates of CC_max_: Using more complex, harder-to-estimate probability distributions would yield the same result. For this reason, we are confident that our bootstrapping procedure, while slightly different from that of Schoppe et al., is comparable to their method.

### Comparison with other models

We compared the results obtained from CNN2 to those of a variety of other models. In implementing our comparisons we used identical cross-validation protocols to determine the training and evaluation data that were used to train CNN2. When the models contained hyperparameters (including regularization parameters), these parameters were optimized on data from the same experimental session used to optimize the hyperparameters of CNN2. We also evaluated all models in the same way, using the normalized Pearson correlation between predicted and actual neural firing rates.

We organized our models for comparison in two broad groups: models that are fully data driven, where all the model parameters were learned from our neural-activity data sets, and models where only a linear regression is performed on neural-activity data sets using regularization by the least absolute shrinkage and selection operator (LASSO). The models using LASSO regression, denoted “trained with regression,” often use external information about visual processing. The fully data-driven models are denoted “trained in TensorFlow.” Our pixel model could fit into either category, but is grouped with the LASSO models. The LASSO comparison models are pixels, SAILnet, Berkeley Wavelet Transform, and five VGG-16-based models. The fully data-driven comparison models are linear–nonlinear (LN), LN-LN, and a one- and a three-convolutional-layer network.

### Pixels

First we constructed a linear model by performing a weighted sum over all pixel values of an image stimulus with a bias to yield a predicted neural activity for each neuron. That is, we formed a prediction

\newcommand{\bialpha}{\boldsymbol{\alpha}}\newcommand{\bibeta}{\boldsymbol{\beta}}\newcommand{\bigamma}{\boldsymbol{\gamma}}\newcommand{\bidelta}{\boldsymbol{\delta}}\newcommand{\bivarepsilon}{\boldsymbol{\varepsilon}}\newcommand{\bizeta}{\boldsymbol{\zeta}}\newcommand{\bieta}{\boldsymbol{\eta}}\newcommand{\bitheta}{\boldsymbol{\theta}}\newcommand{\biiota}{\boldsymbol{\iota}}\newcommand{\bikappa}{\boldsymbol{\kappa}}\newcommand{\bilambda}{\boldsymbol{\lambda}}\newcommand{\bimu}{\boldsymbol{\mu}}\newcommand{\binu}{\boldsymbol{\nu}}\newcommand{\bixi}{\boldsymbol{\xi}}\newcommand{\biomicron}{\boldsymbol{\micron}}\newcommand{\bipi}{\boldsymbol{\pi}}\newcommand{\birho}{\boldsymbol{\rho}}\newcommand{\bisigma}{\boldsymbol{\sigma}}\newcommand{\bitau}{\boldsymbol{\tau}}\newcommand{\biupsilon}{\boldsymbol{\upsilon}}\newcommand{\biphi}{\boldsymbol{\phi}}\newcommand{\bichi}{\boldsymbol{\chi}}\newcommand{\bipsi}{\boldsymbol{\psi}}\newcommand{\biomega}{\boldsymbol{\omega}}\begin{equation}\tag{3}y_{n,i}^{{\rm{pixels}}} = {b_n} + \sum\limits_j {{W_{n,j}}} {x_{j,i}}\end{equation}

for the activity *A_n_*_,_*_i_* of neuron *n*, where *x_j_*_,_*_i_* is the *j*th pixel value in image *i* and the constants *W_n_*_,_*_j_* and *b_n_* are determined from linear regression using LASSO regularization, a type of L1 (sparse) regularized linear regression. The LASSO regularization parameter was optimized on data from the same experimental session used to optimize the hyperparameters of CNN2. Then, leaving this term fixed, we evaluated the model using cross-validation on data from the other nine experimental sessions.

### SAILnet

Next we constructed a SAILnet implementation of a sparse-coding model. In the SAILnet model the images are first whitened, using the whitening filter defined by Olshausen and Field (1996). The whitened images are then passed into a sparse-coding model, which outputs the activations of 1,089 different image features; the number of features is chosen to match the number of pixels. The image features, and the activations, are optimized so as to maximize the fidelity of image encoding while minimizing the number of active features. As an alternative to the SparseNet implementation (Olshausen & Field, 1996), we used the SAILnet model (Zylberberg, Murphy, & DeWeese, 2011).^2^

After training SAILnet on whitened natural-image patches, we froze the weights and passed in whitened versions of the images shown to the monkeys, to obtain the activations *z_j_*_,_*_i_* of each feature (indexed by *j*) for each image (indexed by *i*). We then constructed a linear predictor of the neuron firing rate, from the activations of the sparse-coding features, with prediction

\newcommand{\bialpha}{\boldsymbol{\alpha}}\newcommand{\bibeta}{\boldsymbol{\beta}}\newcommand{\bigamma}{\boldsymbol{\gamma}}\newcommand{\bidelta}{\boldsymbol{\delta}}\newcommand{\bivarepsilon}{\boldsymbol{\varepsilon}}\newcommand{\bizeta}{\boldsymbol{\zeta}}\newcommand{\bieta}{\boldsymbol{\eta}}\newcommand{\bitheta}{\boldsymbol{\theta}}\newcommand{\biiota}{\boldsymbol{\iota}}\newcommand{\bikappa}{\boldsymbol{\kappa}}\newcommand{\bilambda}{\boldsymbol{\lambda}}\newcommand{\bimu}{\boldsymbol{\mu}}\newcommand{\binu}{\boldsymbol{\nu}}\newcommand{\bixi}{\boldsymbol{\xi}}\newcommand{\biomicron}{\boldsymbol{\micron}}\newcommand{\bipi}{\boldsymbol{\pi}}\newcommand{\birho}{\boldsymbol{\rho}}\newcommand{\bisigma}{\boldsymbol{\sigma}}\newcommand{\bitau}{\boldsymbol{\tau}}\newcommand{\biupsilon}{\boldsymbol{\upsilon}}\newcommand{\biphi}{\boldsymbol{\phi}}\newcommand{\bichi}{\boldsymbol{\chi}}\newcommand{\bipsi}{\boldsymbol{\psi}}\newcommand{\biomega}{\boldsymbol{\omega}}\begin{equation}\tag{4}y_{n,i}^{{\rm{SAILnet}}} = {b_n} + \sum\limits_j {{W_{n,j}}} {z_{j,i}}.\end{equation}

Similar to the pixels model, we optimized the biases and weights of this predictor using linear regression with LASSO regularization.

### Berkeley Wavelet Transform

We constructed a Gabor model called the Berkeley Wavelet Transform (BWT) model. To construct the BWT model, we trimmed the outer edges of the small images by cropping the images down to 243 × 243 pixels, removing part of the gray background (the BWT requires square images with edge sizes of a power of 3). We then passed each image through the BWT using code shared by the authors (Willmore, Prenger, Wu, & Gallant, 2008). We did this for all of the small images and then selected those wavelets whose outputs had nonzero variance over the set of images (there are 16,478 of those, out of the total of 59,049 wavelets); the ones with zero variance occurred because they look at the gray parts of the images (see Figure 1B). We used the coefficients of these 16,478 wavelets to predict the neurons' mean firing rates, using LASSO regression with an identical protocol to that of the SAILnet model. The regression was on the weights *W* and biases *b* according to Equation 4, where the variables *z_i_*_,_*_j_* are BWT wavelet activations.

### VGG

To add a comparison to the work (Cadena et al., 2017), we constructed five models from a deep CNN called VGG-16 that has been pretrained on an image-classification task (Simonyan & Zisserman, 2014). We constructed these models out of the activations of VGG at five different depths along the deep network in response to our image set. To do this, we trimmed the outer edges of the small images and cropped down to 224 × 224 pixels, then copied the grayscale images into each of the R, G, and B channels to match the 224 × 224 × 3 input size of VGG. (This duplicates the fact that the monkey has the three input channels but saw grayscale images.) We then passed these images through the (already trained) VGG-16 model and extracted the activations from each layer. Of the layers, we focused on convolutional blocks 2 and 3 because the LASSO fitting is much slower on such large inputs (e.g., >590,000 units in convolutional 3 block 2), and Cadena et al. (2017) show that these blocks provide the best predictions of V1 firing rates. For each layer's activations, we selected those units whose activations had nonzero variance over the set of images; the ones with zero variance occurred because they look at the gray parts of the images. We used the activations of these units to predict the neurons' mean firing rates, using L1-regularized (sparse) LASSO regression. The regression is on the weights *W* and biases *b* according to Equation 4, where the variables *z_i_*_,_*_j_* are VGG activations within the given layer. The five VGG layers we considered are Conv2,1, Conv2,2, Conv3,1, Conv3,2, and Conv3,3 (where Conv*a*,*b* denotes convolutional layer *b* within block *a*).

### LN

We constructed an LN model by applying a nonlinearity to a linear model to yield a prediction for each neuron. According to the LN model we formed a prediction

\newcommand{\bialpha}{\boldsymbol{\alpha}}\newcommand{\bibeta}{\boldsymbol{\beta}}\newcommand{\bigamma}{\boldsymbol{\gamma}}\newcommand{\bidelta}{\boldsymbol{\delta}}\newcommand{\bivarepsilon}{\boldsymbol{\varepsilon}}\newcommand{\bizeta}{\boldsymbol{\zeta}}\newcommand{\bieta}{\boldsymbol{\eta}}\newcommand{\bitheta}{\boldsymbol{\theta}}\newcommand{\biiota}{\boldsymbol{\iota}}\newcommand{\bikappa}{\boldsymbol{\kappa}}\newcommand{\bilambda}{\boldsymbol{\lambda}}\newcommand{\bimu}{\boldsymbol{\mu}}\newcommand{\binu}{\boldsymbol{\nu}}\newcommand{\bixi}{\boldsymbol{\xi}}\newcommand{\biomicron}{\boldsymbol{\micron}}\newcommand{\bipi}{\boldsymbol{\pi}}\newcommand{\birho}{\boldsymbol{\rho}}\newcommand{\bisigma}{\boldsymbol{\sigma}}\newcommand{\bitau}{\boldsymbol{\tau}}\newcommand{\biupsilon}{\boldsymbol{\upsilon}}\newcommand{\biphi}{\boldsymbol{\phi}}\newcommand{\bichi}{\boldsymbol{\chi}}\newcommand{\bipsi}{\boldsymbol{\psi}}\newcommand{\biomega}{\boldsymbol{\omega}}\begin{equation}\tag{5}y_{n,i}^{{\rm{LN}}} = \sigma ({b_n} + \sum\limits_j {{W_{n,j}}} {x_{j,i}})\end{equation}

for the activity of neuron *n*, where *σ*(*x*) is a nonlinear function. A parametric rectified linear was chosen as the nonlinearity because it outperformed a parameterized sigmoid. The parameters of the model were trained in TensorFlow using the same learning process as for the convolutional models, with early stopping as the primary form of regularization.

### LN-LN

We constructed an LN-LN model by cascading two LN models. Thus,

\newcommand{\bialpha}{\boldsymbol{\alpha}}\newcommand{\bibeta}{\boldsymbol{\beta}}\newcommand{\bigamma}{\boldsymbol{\gamma}}\newcommand{\bidelta}{\boldsymbol{\delta}}\newcommand{\bivarepsilon}{\boldsymbol{\varepsilon}}\newcommand{\bizeta}{\boldsymbol{\zeta}}\newcommand{\bieta}{\boldsymbol{\eta}}\newcommand{\bitheta}{\boldsymbol{\theta}}\newcommand{\biiota}{\boldsymbol{\iota}}\newcommand{\bikappa}{\boldsymbol{\kappa}}\newcommand{\bilambda}{\boldsymbol{\lambda}}\newcommand{\bimu}{\boldsymbol{\mu}}\newcommand{\binu}{\boldsymbol{\nu}}\newcommand{\bixi}{\boldsymbol{\xi}}\newcommand{\biomicron}{\boldsymbol{\micron}}\newcommand{\bipi}{\boldsymbol{\pi}}\newcommand{\birho}{\boldsymbol{\rho}}\newcommand{\bisigma}{\boldsymbol{\sigma}}\newcommand{\bitau}{\boldsymbol{\tau}}\newcommand{\biupsilon}{\boldsymbol{\upsilon}}\newcommand{\biphi}{\boldsymbol{\phi}}\newcommand{\bichi}{\boldsymbol{\chi}}\newcommand{\bipsi}{\boldsymbol{\psi}}\newcommand{\biomega}{\boldsymbol{\omega}}\begin{equation}\tag{6}y_{n,i}^{{\rm{LN{\mbox{-}}LN}}} = \sigma \left( {b_n^{(2)} + \sum\limits_k {W_{n,k}^{(2)}} \sigma \left(b_k^{(1)} + \sum\limits_j {W_{k,j}^{(1)}} {x_{j,i}}\right)} \right)\end{equation}

forms the LN-LN model, where *σ*(·) is the rectified linear function, and the superscripts *ℓ* on $\newcommand{\bialpha}{\boldsymbol{\alpha}}\newcommand{\bibeta}{\boldsymbol{\beta}}\newcommand{\bigamma}{\boldsymbol{\gamma}}\newcommand{\bidelta}{\boldsymbol{\delta}}\newcommand{\bivarepsilon}{\boldsymbol{\varepsilon}}\newcommand{\bizeta}{\boldsymbol{\zeta}}\newcommand{\bieta}{\boldsymbol{\eta}}\newcommand{\bitheta}{\boldsymbol{\theta}}\newcommand{\biiota}{\boldsymbol{\iota}}\newcommand{\bikappa}{\boldsymbol{\kappa}}\newcommand{\bilambda}{\boldsymbol{\lambda}}\newcommand{\bimu}{\boldsymbol{\mu}}\newcommand{\binu}{\boldsymbol{\nu}}\newcommand{\bixi}{\boldsymbol{\xi}}\newcommand{\biomicron}{\boldsymbol{\micron}}\newcommand{\bipi}{\boldsymbol{\pi}}\newcommand{\birho}{\boldsymbol{\rho}}\newcommand{\bisigma}{\boldsymbol{\sigma}}\newcommand{\bitau}{\boldsymbol{\tau}}\newcommand{\biupsilon}{\boldsymbol{\upsilon}}\newcommand{\biphi}{\boldsymbol{\phi}}\newcommand{\bichi}{\boldsymbol{\chi}}\newcommand{\bipsi}{\boldsymbol{\psi}}\newcommand{\biomega}{\boldsymbol{\omega}}{W^{(\ell )}}$ and $\newcommand{\bialpha}{\boldsymbol{\alpha}}\newcommand{\bibeta}{\boldsymbol{\beta}}\newcommand{\bigamma}{\boldsymbol{\gamma}}\newcommand{\bidelta}{\boldsymbol{\delta}}\newcommand{\bivarepsilon}{\boldsymbol{\varepsilon}}\newcommand{\bizeta}{\boldsymbol{\zeta}}\newcommand{\bieta}{\boldsymbol{\eta}}\newcommand{\bitheta}{\boldsymbol{\theta}}\newcommand{\biiota}{\boldsymbol{\iota}}\newcommand{\bikappa}{\boldsymbol{\kappa}}\newcommand{\bilambda}{\boldsymbol{\lambda}}\newcommand{\bimu}{\boldsymbol{\mu}}\newcommand{\binu}{\boldsymbol{\nu}}\newcommand{\bixi}{\boldsymbol{\xi}}\newcommand{\biomicron}{\boldsymbol{\micron}}\newcommand{\bipi}{\boldsymbol{\pi}}\newcommand{\birho}{\boldsymbol{\rho}}\newcommand{\bisigma}{\boldsymbol{\sigma}}\newcommand{\bitau}{\boldsymbol{\tau}}\newcommand{\biupsilon}{\boldsymbol{\upsilon}}\newcommand{\biphi}{\boldsymbol{\phi}}\newcommand{\bichi}{\boldsymbol{\chi}}\newcommand{\bipsi}{\boldsymbol{\psi}}\newcommand{\biomega}{\boldsymbol{\omega}}{b^{(\ell )}}$ denote the layer. This model was trained in TensorFlow using the same learning process as the convolutional models, with early stopping as the primary form of regularization. Its hyperparameters, such as the number of hidden elements, were optimized on the same experimental session as CNN2. Our LN-LN model is a nonconvolutional LN-LN. There are more complex versions that use convolutions and pooling at the input stage; those are more similar to our CNN1 (Vintch, Movshon, & Simoncelli, 2015).

### CNN1 and CNN3

In order to show the importance of model depth or lack thereof, we compared our chosen best model—the two-convolutional-layer network (CNN2)—to a single-convolutional-layer network (CNN1) and a three-convolutional-layer network (CNN3). The hyperparameters of CNN1 and CNN3 were optimized on data from the same experimental session used to optimize CNN2, and the models were regularized using a combination of dropout and early stopping.

### Characterizing the selectivity of cells

To show that our model describes the activity of a broad class of cell types, we grouped the cells into functional classes and looked at how well the firing rates from each class could be predicted by our neural-network model. We classified cells by their selectivity to specific natural images, their selectivity to specific orientations of grating stimuli, their average firing rate over all images *A*, and their reliability CC_max_.

The selectivity of each neuron to specific natural images is quantified by

\newcommand{\bialpha}{\boldsymbol{\alpha}}\newcommand{\bibeta}{\boldsymbol{\beta}}\newcommand{\bigamma}{\boldsymbol{\gamma}}\newcommand{\bidelta}{\boldsymbol{\delta}}\newcommand{\bivarepsilon}{\boldsymbol{\varepsilon}}\newcommand{\bizeta}{\boldsymbol{\zeta}}\newcommand{\bieta}{\boldsymbol{\eta}}\newcommand{\bitheta}{\boldsymbol{\theta}}\newcommand{\biiota}{\boldsymbol{\iota}}\newcommand{\bikappa}{\boldsymbol{\kappa}}\newcommand{\bilambda}{\boldsymbol{\lambda}}\newcommand{\bimu}{\boldsymbol{\mu}}\newcommand{\binu}{\boldsymbol{\nu}}\newcommand{\bixi}{\boldsymbol{\xi}}\newcommand{\biomicron}{\boldsymbol{\micron}}\newcommand{\bipi}{\boldsymbol{\pi}}\newcommand{\birho}{\boldsymbol{\rho}}\newcommand{\bisigma}{\boldsymbol{\sigma}}\newcommand{\bitau}{\boldsymbol{\tau}}\newcommand{\biupsilon}{\boldsymbol{\upsilon}}\newcommand{\biphi}{\boldsymbol{\phi}}\newcommand{\bichi}{\boldsymbol{\chi}}\newcommand{\bipsi}{\boldsymbol{\psi}}\newcommand{\biomega}{\boldsymbol{\omega}}\begin{equation}\tag{7}{\rm{image\ selectivity\ index}} = \left( {{{N}} - {{{{(\sum\nolimits_{{i}} {{{{A}}_{{i}}}} )}^{2}}} \over {\sum\nolimits_{{i}} {{{(A}}_{{i}}^{2})} }}} \right){{1} \over {{{N}} - {1}}},\end{equation}

where *A_i_* is the cell's firing rate indexed *i* over the set of *N* images (Zylberberg & DeWeese, 2013). This index has a value of 0 for neurons that fire equally to all images and a value of 1 for cells that spike in response to only one of the images.

The neuron's orientation selectivity is measured by

\newcommand{\bialpha}{\boldsymbol{\alpha}}\newcommand{\bibeta}{\boldsymbol{\beta}}\newcommand{\bigamma}{\boldsymbol{\gamma}}\newcommand{\bidelta}{\boldsymbol{\delta}}\newcommand{\bivarepsilon}{\boldsymbol{\varepsilon}}\newcommand{\bizeta}{\boldsymbol{\zeta}}\newcommand{\bieta}{\boldsymbol{\eta}}\newcommand{\bitheta}{\boldsymbol{\theta}}\newcommand{\biiota}{\boldsymbol{\iota}}\newcommand{\bikappa}{\boldsymbol{\kappa}}\newcommand{\bilambda}{\boldsymbol{\lambda}}\newcommand{\bimu}{\boldsymbol{\mu}}\newcommand{\binu}{\boldsymbol{\nu}}\newcommand{\bixi}{\boldsymbol{\xi}}\newcommand{\biomicron}{\boldsymbol{\micron}}\newcommand{\bipi}{\boldsymbol{\pi}}\newcommand{\birho}{\boldsymbol{\rho}}\newcommand{\bisigma}{\boldsymbol{\sigma}}\newcommand{\bitau}{\boldsymbol{\tau}}\newcommand{\biupsilon}{\boldsymbol{\upsilon}}\newcommand{\biphi}{\boldsymbol{\phi}}\newcommand{\bichi}{\boldsymbol{\chi}}\newcommand{\bipsi}{\boldsymbol{\psi}}\newcommand{\biomega}{\boldsymbol{\omega}}\begin{equation}\tag{8}{\rm{circular\ variance}} = 1 - {{\left| {\sum\nolimits_\theta {{{{A}}_\theta }} {{{e}}^{{{i2}}\theta }}} \right|} \over {\sum\nolimits_\theta {{{{A}}_\theta }} }},\end{equation}

where *A_θ_* is the neuron's firing rate in response to a grating oriented at angle *θ*. The circular variance is less sensitive to noise than the more commonly used orientation-selectivity index (Mazurek, Kager, & Van Hooser, 2014). Following the results of Mazurek et al. we used thresholds of circular variance < 0.6 to define orientation-selective cells (the simple and complex cells according to Hubel & Wiesel, 1959) and circular variance > 0.75 non-orientation-selective cells. We omitted all other cells from these two groupings.

## Results

**Figure 4:**
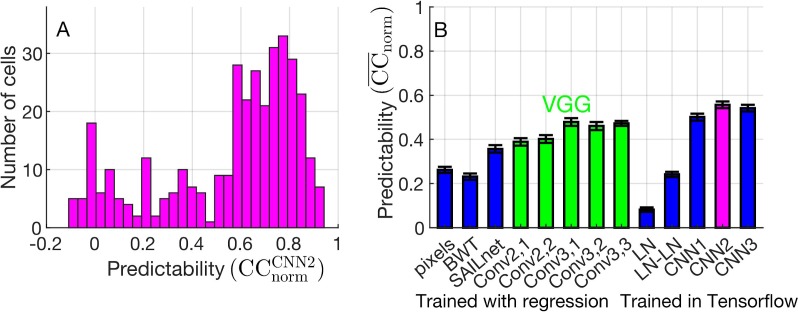
The performance of the best convolutional network model, CNN2. (A) A histogram of the normalized Pearson correlation coefficients between the network predictions and the actual firing rates $\newcommand{\bialpha}{\boldsymbol{\alpha}}\newcommand{\bibeta}{\boldsymbol{\beta}}\newcommand{\bigamma}{\boldsymbol{\gamma}}\newcommand{\bidelta}{\boldsymbol{\delta}}\newcommand{\bivarepsilon}{\boldsymbol{\varepsilon}}\newcommand{\bizeta}{\boldsymbol{\zeta}}\newcommand{\bieta}{\boldsymbol{\eta}}\newcommand{\bitheta}{\boldsymbol{\theta}}\newcommand{\biiota}{\boldsymbol{\iota}}\newcommand{\bikappa}{\boldsymbol{\kappa}}\newcommand{\bilambda}{\boldsymbol{\lambda}}\newcommand{\bimu}{\boldsymbol{\mu}}\newcommand{\binu}{\boldsymbol{\nu}}\newcommand{\bixi}{\boldsymbol{\xi}}\newcommand{\biomicron}{\boldsymbol{\micron}}\newcommand{\bipi}{\boldsymbol{\pi}}\newcommand{\birho}{\boldsymbol{\rho}}\newcommand{\bisigma}{\boldsymbol{\sigma}}\newcommand{\bitau}{\boldsymbol{\tau}}\newcommand{\biupsilon}{\boldsymbol{\upsilon}}\newcommand{\biphi}{\boldsymbol{\phi}}\newcommand{\bichi}{\boldsymbol{\chi}}\newcommand{\bipsi}{\boldsymbol{\psi}}\newcommand{\biomega}{\boldsymbol{\omega}}{\rm{CC}}_{{\rm{norm}}}^{{\rm{CNN2}}}$ of all 355 neurons. (B) The average performance of the convolution-neural-network predictor (CNN2) compared to a variety of other models. The models are grouped as models that are trained only with regularized linear regression by least absolute shrinkage and selection operator on the neural-activity data (pixels, Berkeley Wavelet Transform [BWT], SAILnet, and our VGG models) and models where all the parameters are fully trained on the neural activity using TensorFlow (linear–nonlinear [LN], LN-LN, CNN1, and CNN2). The five VGG models in green are denoted Conva,b for convolutional layer b within block a.

Using our optimal network, we predicted firing rates that were highly correlated with the measured firing rates for most neurons (Figure 4A) when evaluated on held-out data. The correlation between the predicted and actual neural firing rates is $\newcommand{\bialpha}{\boldsymbol{\alpha}}\newcommand{\bibeta}{\boldsymbol{\beta}}\newcommand{\bigamma}{\boldsymbol{\gamma}}\newcommand{\bidelta}{\boldsymbol{\delta}}\newcommand{\bivarepsilon}{\boldsymbol{\varepsilon}}\newcommand{\bizeta}{\boldsymbol{\zeta}}\newcommand{\bieta}{\boldsymbol{\eta}}\newcommand{\bitheta}{\boldsymbol{\theta}}\newcommand{\biiota}{\boldsymbol{\iota}}\newcommand{\bikappa}{\boldsymbol{\kappa}}\newcommand{\bilambda}{\boldsymbol{\lambda}}\newcommand{\bimu}{\boldsymbol{\mu}}\newcommand{\binu}{\boldsymbol{\nu}}\newcommand{\bixi}{\boldsymbol{\xi}}\newcommand{\biomicron}{\boldsymbol{\micron}}\newcommand{\bipi}{\boldsymbol{\pi}}\newcommand{\birho}{\boldsymbol{\rho}}\newcommand{\bisigma}{\boldsymbol{\sigma}}\newcommand{\bitau}{\boldsymbol{\tau}}\newcommand{\biupsilon}{\boldsymbol{\upsilon}}\newcommand{\biphi}{\boldsymbol{\phi}}\newcommand{\bichi}{\boldsymbol{\chi}}\newcommand{\bipsi}{\boldsymbol{\psi}}\newcommand{\biomega}{\boldsymbol{\omega}}\overline {{\rm{CC}}} _{{\rm{norm}}}^{{\rm{CNN2}}} = {0}.{556}\ \pm\ {0}.{015}$ ($\newcommand{\bialpha}{\boldsymbol{\alpha}}\newcommand{\bibeta}{\boldsymbol{\beta}}\newcommand{\bigamma}{\boldsymbol{\gamma}}\newcommand{\bidelta}{\boldsymbol{\delta}}\newcommand{\bivarepsilon}{\boldsymbol{\varepsilon}}\newcommand{\bizeta}{\boldsymbol{\zeta}}\newcommand{\bieta}{\boldsymbol{\eta}}\newcommand{\bitheta}{\boldsymbol{\theta}}\newcommand{\biiota}{\boldsymbol{\iota}}\newcommand{\bikappa}{\boldsymbol{\kappa}}\newcommand{\bilambda}{\boldsymbol{\lambda}}\newcommand{\bimu}{\boldsymbol{\mu}}\newcommand{\binu}{\boldsymbol{\nu}}\newcommand{\bixi}{\boldsymbol{\xi}}\newcommand{\biomicron}{\boldsymbol{\micron}}\newcommand{\bipi}{\boldsymbol{\pi}}\newcommand{\birho}{\boldsymbol{\rho}}\newcommand{\bisigma}{\boldsymbol{\sigma}}\newcommand{\bitau}{\boldsymbol{\tau}}\newcommand{\biupsilon}{\boldsymbol{\upsilon}}\newcommand{\biphi}{\boldsymbol{\phi}}\newcommand{\bichi}{\boldsymbol{\chi}}\newcommand{\bipsi}{\boldsymbol{\psi}}\newcommand{\biomega}{\boldsymbol{\omega}}\overline {{\rm{CC}}} _{\rm abs}^{{\rm{CNN2}}} = 0.493\ \pm\ 0.014$) averaged over all 355 neurons in the evaluation set without using any selection criteria (Figure 4B). To benchmark the accuracy of our model, we compared it to a variety of other models (Figure 4B). We found that CNN2 is, indeed, the best-performing model. In comparison with fully data-driven models (denoted “trained in TensorFlow”), we found that our two-convolutional-layer CNN2 is more accurate than single- (CNN1) and triple-convolutional-layer (CNN3) models, and far more accurate than shallower models such as LN. Compared to pretrained models where only LASSO regression was performed on the neural-activation data, we found that our optimized data-driven CNN outperforms models based on VGG, the Berkeley Wavelet Transform, and the SAILnet sparse-coding algorithm (see Methods for details).

Because simple and complex cells have been extensively studied, we were motivated to compare the predictability of simple and complex cells to the predictability of the other neurons in the data set. Grouping the cells into orientation-selective (simple- and complexlike cells) and non-orientation-selective cells (see Methods), we found that our network predicts non-orientation-selective cell responses with $\newcommand{\bialpha}{\boldsymbol{\alpha}}\newcommand{\bibeta}{\boldsymbol{\beta}}\newcommand{\bigamma}{\boldsymbol{\gamma}}\newcommand{\bidelta}{\boldsymbol{\delta}}\newcommand{\bivarepsilon}{\boldsymbol{\varepsilon}}\newcommand{\bizeta}{\boldsymbol{\zeta}}\newcommand{\bieta}{\boldsymbol{\eta}}\newcommand{\bitheta}{\boldsymbol{\theta}}\newcommand{\biiota}{\boldsymbol{\iota}}\newcommand{\bikappa}{\boldsymbol{\kappa}}\newcommand{\bilambda}{\boldsymbol{\lambda}}\newcommand{\bimu}{\boldsymbol{\mu}}\newcommand{\binu}{\boldsymbol{\nu}}\newcommand{\bixi}{\boldsymbol{\xi}}\newcommand{\biomicron}{\boldsymbol{\micron}}\newcommand{\bipi}{\boldsymbol{\pi}}\newcommand{\birho}{\boldsymbol{\rho}}\newcommand{\bisigma}{\boldsymbol{\sigma}}\newcommand{\bitau}{\boldsymbol{\tau}}\newcommand{\biupsilon}{\boldsymbol{\upsilon}}\newcommand{\biphi}{\boldsymbol{\phi}}\newcommand{\bichi}{\boldsymbol{\chi}}\newcommand{\bipsi}{\boldsymbol{\psi}}\newcommand{\biomega}{\boldsymbol{\omega}}\overline {{\rm{CC}}} _{{\rm{norm}}}^{{\rm{CNN2}}} = {0}.{50}\ \pm\ {0}.{02}$ and orientation-selective cell responses with $\newcommand{\bialpha}{\boldsymbol{\alpha}}\newcommand{\bibeta}{\boldsymbol{\beta}}\newcommand{\bigamma}{\boldsymbol{\gamma}}\newcommand{\bidelta}{\boldsymbol{\delta}}\newcommand{\bivarepsilon}{\boldsymbol{\varepsilon}}\newcommand{\bizeta}{\boldsymbol{\zeta}}\newcommand{\bieta}{\boldsymbol{\eta}}\newcommand{\bitheta}{\boldsymbol{\theta}}\newcommand{\biiota}{\boldsymbol{\iota}}\newcommand{\bikappa}{\boldsymbol{\kappa}}\newcommand{\bilambda}{\boldsymbol{\lambda}}\newcommand{\bimu}{\boldsymbol{\mu}}\newcommand{\binu}{\boldsymbol{\nu}}\newcommand{\bixi}{\boldsymbol{\xi}}\newcommand{\biomicron}{\boldsymbol{\micron}}\newcommand{\bipi}{\boldsymbol{\pi}}\newcommand{\birho}{\boldsymbol{\rho}}\newcommand{\bisigma}{\boldsymbol{\sigma}}\newcommand{\bitau}{\boldsymbol{\tau}}\newcommand{\biupsilon}{\boldsymbol{\upsilon}}\newcommand{\biphi}{\boldsymbol{\phi}}\newcommand{\bichi}{\boldsymbol{\chi}}\newcommand{\bipsi}{\boldsymbol{\psi}}\newcommand{\biomega}{\boldsymbol{\omega}}\overline {{\rm{CC}}} _{{\rm{norm}}}^{{\rm{CNN2}}} = {0}.{55}\ \pm\ {0}.{04}$. Therefore, our model predicts the firing rates of both cell types, performing slightly better on the simple- and complexlike cells than the non-orientation-selective cells.

**Figure 5:**
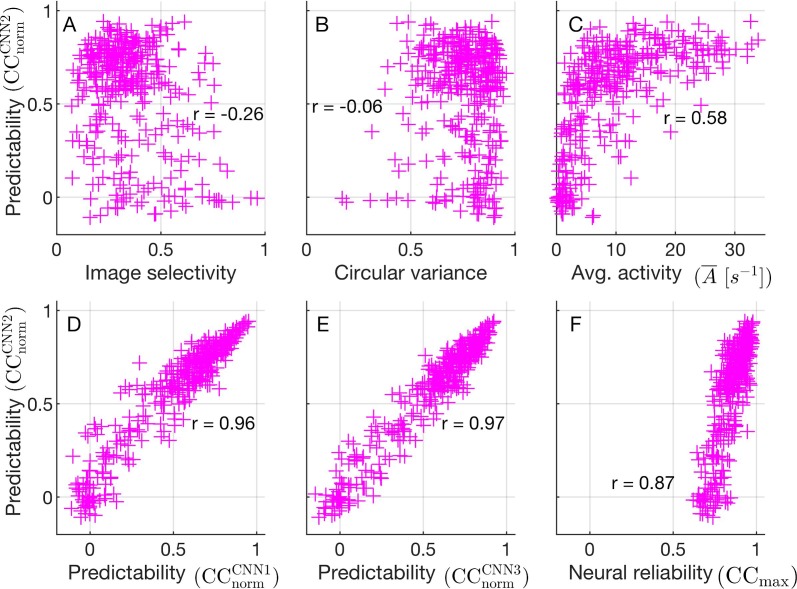
Characterizing the predictability of CNN2 ($\newcommand{\bialpha}{\boldsymbol{\alpha}}\newcommand{\bibeta}{\boldsymbol{\beta}}\newcommand{\bigamma}{\boldsymbol{\gamma}}\newcommand{\bidelta}{\boldsymbol{\delta}}\newcommand{\bivarepsilon}{\boldsymbol{\varepsilon}}\newcommand{\bizeta}{\boldsymbol{\zeta}}\newcommand{\bieta}{\boldsymbol{\eta}}\newcommand{\bitheta}{\boldsymbol{\theta}}\newcommand{\biiota}{\boldsymbol{\iota}}\newcommand{\bikappa}{\boldsymbol{\kappa}}\newcommand{\bilambda}{\boldsymbol{\lambda}}\newcommand{\bimu}{\boldsymbol{\mu}}\newcommand{\binu}{\boldsymbol{\nu}}\newcommand{\bixi}{\boldsymbol{\xi}}\newcommand{\biomicron}{\boldsymbol{\micron}}\newcommand{\bipi}{\boldsymbol{\pi}}\newcommand{\birho}{\boldsymbol{\rho}}\newcommand{\bisigma}{\boldsymbol{\sigma}}\newcommand{\bitau}{\boldsymbol{\tau}}\newcommand{\biupsilon}{\boldsymbol{\upsilon}}\newcommand{\biphi}{\boldsymbol{\phi}}\newcommand{\bichi}{\boldsymbol{\chi}}\newcommand{\bipsi}{\boldsymbol{\psi}}\newcommand{\biomega}{\boldsymbol{\omega}}{\rm{CC}}_{{\rm{norm}}}^{{\rm{CNN2}}}$) over the population of neurons; each data point corresponds to a single neuron. (A) Scatterplot of how well the predictor can predict each neuron's firing rate $\newcommand{\bialpha}{\boldsymbol{\alpha}}\newcommand{\bibeta}{\boldsymbol{\beta}}\newcommand{\bigamma}{\boldsymbol{\gamma}}\newcommand{\bidelta}{\boldsymbol{\delta}}\newcommand{\bivarepsilon}{\boldsymbol{\varepsilon}}\newcommand{\bizeta}{\boldsymbol{\zeta}}\newcommand{\bieta}{\boldsymbol{\eta}}\newcommand{\bitheta}{\boldsymbol{\theta}}\newcommand{\biiota}{\boldsymbol{\iota}}\newcommand{\bikappa}{\boldsymbol{\kappa}}\newcommand{\bilambda}{\boldsymbol{\lambda}}\newcommand{\bimu}{\boldsymbol{\mu}}\newcommand{\binu}{\boldsymbol{\nu}}\newcommand{\bixi}{\boldsymbol{\xi}}\newcommand{\biomicron}{\boldsymbol{\micron}}\newcommand{\bipi}{\boldsymbol{\pi}}\newcommand{\birho}{\boldsymbol{\rho}}\newcommand{\bisigma}{\boldsymbol{\sigma}}\newcommand{\bitau}{\boldsymbol{\tau}}\newcommand{\biupsilon}{\boldsymbol{\upsilon}}\newcommand{\biphi}{\boldsymbol{\phi}}\newcommand{\bichi}{\boldsymbol{\chi}}\newcommand{\bipsi}{\boldsymbol{\psi}}\newcommand{\biomega}{\boldsymbol{\omega}}{\rm{CC}}_{{\rm{norm}}}^{{\rm{CNN2}}}$ (vertical axis) against the neuron's image selectivity (horizontal axis). (B) Scatterplot against the neuron's circular variance (horizontal axis). (C) Scatterplot against the neuron's average firing rate $\newcommand{\bialpha}{\boldsymbol{\alpha}}\newcommand{\bibeta}{\boldsymbol{\beta}}\newcommand{\bigamma}{\boldsymbol{\gamma}}\newcommand{\bidelta}{\boldsymbol{\delta}}\newcommand{\bivarepsilon}{\boldsymbol{\varepsilon}}\newcommand{\bizeta}{\boldsymbol{\zeta}}\newcommand{\bieta}{\boldsymbol{\eta}}\newcommand{\bitheta}{\boldsymbol{\theta}}\newcommand{\biiota}{\boldsymbol{\iota}}\newcommand{\bikappa}{\boldsymbol{\kappa}}\newcommand{\bilambda}{\boldsymbol{\lambda}}\newcommand{\bimu}{\boldsymbol{\mu}}\newcommand{\binu}{\boldsymbol{\nu}}\newcommand{\bixi}{\boldsymbol{\xi}}\newcommand{\biomicron}{\boldsymbol{\micron}}\newcommand{\bipi}{\boldsymbol{\pi}}\newcommand{\birho}{\boldsymbol{\rho}}\newcommand{\bisigma}{\boldsymbol{\sigma}}\newcommand{\bitau}{\boldsymbol{\tau}}\newcommand{\biupsilon}{\boldsymbol{\upsilon}}\newcommand{\biphi}{\boldsymbol{\phi}}\newcommand{\bichi}{\boldsymbol{\chi}}\newcommand{\bipsi}{\boldsymbol{\psi}}\newcommand{\biomega}{\boldsymbol{\omega}}\bar A$ (horizontal axis). (D) Scatterplot against the predictability $\newcommand{\bialpha}{\boldsymbol{\alpha}}\newcommand{\bibeta}{\boldsymbol{\beta}}\newcommand{\bigamma}{\boldsymbol{\gamma}}\newcommand{\bidelta}{\boldsymbol{\delta}}\newcommand{\bivarepsilon}{\boldsymbol{\varepsilon}}\newcommand{\bizeta}{\boldsymbol{\zeta}}\newcommand{\bieta}{\boldsymbol{\eta}}\newcommand{\bitheta}{\boldsymbol{\theta}}\newcommand{\biiota}{\boldsymbol{\iota}}\newcommand{\bikappa}{\boldsymbol{\kappa}}\newcommand{\bilambda}{\boldsymbol{\lambda}}\newcommand{\bimu}{\boldsymbol{\mu}}\newcommand{\binu}{\boldsymbol{\nu}}\newcommand{\bixi}{\boldsymbol{\xi}}\newcommand{\biomicron}{\boldsymbol{\micron}}\newcommand{\bipi}{\boldsymbol{\pi}}\newcommand{\birho}{\boldsymbol{\rho}}\newcommand{\bisigma}{\boldsymbol{\sigma}}\newcommand{\bitau}{\boldsymbol{\tau}}\newcommand{\biupsilon}{\boldsymbol{\upsilon}}\newcommand{\biphi}{\boldsymbol{\phi}}\newcommand{\bichi}{\boldsymbol{\chi}}\newcommand{\bipsi}{\boldsymbol{\psi}}\newcommand{\biomega}{\boldsymbol{\omega}}{\rm{CC}}_{{\rm{norm}}}^{{\rm{CNN1}}}$ of CNN1 (horizontal axis). (E) Scatterplot against the predictability $\newcommand{\bialpha}{\boldsymbol{\alpha}}\newcommand{\bibeta}{\boldsymbol{\beta}}\newcommand{\bigamma}{\boldsymbol{\gamma}}\newcommand{\bidelta}{\boldsymbol{\delta}}\newcommand{\bivarepsilon}{\boldsymbol{\varepsilon}}\newcommand{\bizeta}{\boldsymbol{\zeta}}\newcommand{\bieta}{\boldsymbol{\eta}}\newcommand{\bitheta}{\boldsymbol{\theta}}\newcommand{\biiota}{\boldsymbol{\iota}}\newcommand{\bikappa}{\boldsymbol{\kappa}}\newcommand{\bilambda}{\boldsymbol{\lambda}}\newcommand{\bimu}{\boldsymbol{\mu}}\newcommand{\binu}{\boldsymbol{\nu}}\newcommand{\bixi}{\boldsymbol{\xi}}\newcommand{\biomicron}{\boldsymbol{\micron}}\newcommand{\bipi}{\boldsymbol{\pi}}\newcommand{\birho}{\boldsymbol{\rho}}\newcommand{\bisigma}{\boldsymbol{\sigma}}\newcommand{\bitau}{\boldsymbol{\tau}}\newcommand{\biupsilon}{\boldsymbol{\upsilon}}\newcommand{\biphi}{\boldsymbol{\phi}}\newcommand{\bichi}{\boldsymbol{\chi}}\newcommand{\bipsi}{\boldsymbol{\psi}}\newcommand{\biomega}{\boldsymbol{\omega}}{\rm{CC}}_{{\rm{norm}}}^{{\rm{CNN3}}}$ of CNN3 (horizontal axis). (F) Scatterplot against the neural reliability CC_max_ (horizontal axis).

Given that some neurons' firing rates are well predicted by the network (CNN2) while others are not, we were motivated to ask what distinguishes predictable from unpredictable cells. Furthermore, we found that the cells that are well predicted CNN2 are also well predicted by CNN1 (Figure 5D) and CNN3 (Figure 5E), indicating these differences in predictability are set by the cells themselves rather than the neural-network architecture. To better understand what is driving these differences among the cells, we characterized the cells according to several metrics and then saw how these metrics can explain the distribution of predictability over the population of cells. We quantified the cells according to their orientation selectivity (see Methods), their image selectivity (see Methods), their average firing rate over all images and trials $\newcommand{\bialpha}{\boldsymbol{\alpha}}\newcommand{\bibeta}{\boldsymbol{\beta}}\newcommand{\bigamma}{\boldsymbol{\gamma}}\newcommand{\bidelta}{\boldsymbol{\delta}}\newcommand{\bivarepsilon}{\boldsymbol{\varepsilon}}\newcommand{\bizeta}{\boldsymbol{\zeta}}\newcommand{\bieta}{\boldsymbol{\eta}}\newcommand{\bitheta}{\boldsymbol{\theta}}\newcommand{\biiota}{\boldsymbol{\iota}}\newcommand{\bikappa}{\boldsymbol{\kappa}}\newcommand{\bilambda}{\boldsymbol{\lambda}}\newcommand{\bimu}{\boldsymbol{\mu}}\newcommand{\binu}{\boldsymbol{\nu}}\newcommand{\bixi}{\boldsymbol{\xi}}\newcommand{\biomicron}{\boldsymbol{\micron}}\newcommand{\bipi}{\boldsymbol{\pi}}\newcommand{\birho}{\boldsymbol{\rho}}\newcommand{\bisigma}{\boldsymbol{\sigma}}\newcommand{\bitau}{\boldsymbol{\tau}}\newcommand{\biupsilon}{\boldsymbol{\upsilon}}\newcommand{\biphi}{\boldsymbol{\phi}}\newcommand{\bichi}{\boldsymbol{\chi}}\newcommand{\bipsi}{\boldsymbol{\psi}}\newcommand{\biomega}{\boldsymbol{\omega}}\bar A$, and their reliability over repeat image presentations, as quantified by the theoretical upper bound on predictability CC_max_. Comparing the predictability of each cell's firing rates with its respective image-selectivity index (Figure 5A) and circular variance (Figure 5B), we found that the predictability depends only weakly on these characteristics. Thus, orientation selectivity and image selectivity are only minor factors in determining how well our model performs.

We found that a neuron's activation, or mean firing rate over all images $\newcommand{\bialpha}{\boldsymbol{\alpha}}\newcommand{\bibeta}{\boldsymbol{\beta}}\newcommand{\bigamma}{\boldsymbol{\gamma}}\newcommand{\bidelta}{\boldsymbol{\delta}}\newcommand{\bivarepsilon}{\boldsymbol{\varepsilon}}\newcommand{\bizeta}{\boldsymbol{\zeta}}\newcommand{\bieta}{\boldsymbol{\eta}}\newcommand{\bitheta}{\boldsymbol{\theta}}\newcommand{\biiota}{\boldsymbol{\iota}}\newcommand{\bikappa}{\boldsymbol{\kappa}}\newcommand{\bilambda}{\boldsymbol{\lambda}}\newcommand{\bimu}{\boldsymbol{\mu}}\newcommand{\binu}{\boldsymbol{\nu}}\newcommand{\bixi}{\boldsymbol{\xi}}\newcommand{\biomicron}{\boldsymbol{\micron}}\newcommand{\bipi}{\boldsymbol{\pi}}\newcommand{\birho}{\boldsymbol{\rho}}\newcommand{\bisigma}{\boldsymbol{\sigma}}\newcommand{\bitau}{\boldsymbol{\tau}}\newcommand{\biupsilon}{\boldsymbol{\upsilon}}\newcommand{\biphi}{\boldsymbol{\phi}}\newcommand{\bichi}{\boldsymbol{\chi}}\newcommand{\bipsi}{\boldsymbol{\psi}}\newcommand{\biomega}{\boldsymbol{\omega}}\bar A$ (Figure 5C), and its limit neural reliability CC_max_ (Figure 5F) are both strongly related to the model's performance. Cells with a low mean firing rate $\newcommand{\bialpha}{\boldsymbol{\alpha}}\newcommand{\bibeta}{\boldsymbol{\beta}}\newcommand{\bigamma}{\boldsymbol{\gamma}}\newcommand{\bidelta}{\boldsymbol{\delta}}\newcommand{\bivarepsilon}{\boldsymbol{\varepsilon}}\newcommand{\bizeta}{\boldsymbol{\zeta}}\newcommand{\bieta}{\boldsymbol{\eta}}\newcommand{\bitheta}{\boldsymbol{\theta}}\newcommand{\biiota}{\boldsymbol{\iota}}\newcommand{\bikappa}{\boldsymbol{\kappa}}\newcommand{\bilambda}{\boldsymbol{\lambda}}\newcommand{\bimu}{\boldsymbol{\mu}}\newcommand{\binu}{\boldsymbol{\nu}}\newcommand{\bixi}{\boldsymbol{\xi}}\newcommand{\biomicron}{\boldsymbol{\micron}}\newcommand{\bipi}{\boldsymbol{\pi}}\newcommand{\birho}{\boldsymbol{\rho}}\newcommand{\bisigma}{\boldsymbol{\sigma}}\newcommand{\bitau}{\boldsymbol{\tau}}\newcommand{\biupsilon}{\boldsymbol{\upsilon}}\newcommand{\biphi}{\boldsymbol{\phi}}\newcommand{\bichi}{\boldsymbol{\chi}}\newcommand{\bipsi}{\boldsymbol{\psi}}\newcommand{\biomega}{\boldsymbol{\omega}}\bar A \lt 5$ Hz are less well described by our model, with $\newcommand{\bialpha}{\boldsymbol{\alpha}}\newcommand{\bibeta}{\boldsymbol{\beta}}\newcommand{\bigamma}{\boldsymbol{\gamma}}\newcommand{\bidelta}{\boldsymbol{\delta}}\newcommand{\bivarepsilon}{\boldsymbol{\varepsilon}}\newcommand{\bizeta}{\boldsymbol{\zeta}}\newcommand{\bieta}{\boldsymbol{\eta}}\newcommand{\bitheta}{\boldsymbol{\theta}}\newcommand{\biiota}{\boldsymbol{\iota}}\newcommand{\bikappa}{\boldsymbol{\kappa}}\newcommand{\bilambda}{\boldsymbol{\lambda}}\newcommand{\bimu}{\boldsymbol{\mu}}\newcommand{\binu}{\boldsymbol{\nu}}\newcommand{\bixi}{\boldsymbol{\xi}}\newcommand{\biomicron}{\boldsymbol{\micron}}\newcommand{\bipi}{\boldsymbol{\pi}}\newcommand{\birho}{\boldsymbol{\rho}}\newcommand{\bisigma}{\boldsymbol{\sigma}}\newcommand{\bitau}{\boldsymbol{\tau}}\newcommand{\biupsilon}{\boldsymbol{\upsilon}}\newcommand{\biphi}{\boldsymbol{\phi}}\newcommand{\bichi}{\boldsymbol{\chi}}\newcommand{\bipsi}{\boldsymbol{\psi}}\newcommand{\biomega}{\boldsymbol{\omega}}\overline {{\rm{CC}}} _{{\rm{norm}}}^{{\rm{CNN2}}} = 0.29\ \pm\ 0.03$. Selecting only the more active cells ($\newcommand{\bialpha}{\boldsymbol{\alpha}}\newcommand{\bibeta}{\boldsymbol{\beta}}\newcommand{\bigamma}{\boldsymbol{\gamma}}\newcommand{\bidelta}{\boldsymbol{\delta}}\newcommand{\bivarepsilon}{\boldsymbol{\varepsilon}}\newcommand{\bizeta}{\boldsymbol{\zeta}}\newcommand{\bieta}{\boldsymbol{\eta}}\newcommand{\bitheta}{\boldsymbol{\theta}}\newcommand{\biiota}{\boldsymbol{\iota}}\newcommand{\bikappa}{\boldsymbol{\kappa}}\newcommand{\bilambda}{\boldsymbol{\lambda}}\newcommand{\bimu}{\boldsymbol{\mu}}\newcommand{\binu}{\boldsymbol{\nu}}\newcommand{\bixi}{\boldsymbol{\xi}}\newcommand{\biomicron}{\boldsymbol{\micron}}\newcommand{\bipi}{\boldsymbol{\pi}}\newcommand{\birho}{\boldsymbol{\rho}}\newcommand{\bisigma}{\boldsymbol{\sigma}}\newcommand{\bitau}{\boldsymbol{\tau}}\newcommand{\biupsilon}{\boldsymbol{\upsilon}}\newcommand{\biphi}{\boldsymbol{\phi}}\newcommand{\bichi}{\boldsymbol{\chi}}\newcommand{\bipsi}{\boldsymbol{\psi}}\newcommand{\biomega}{\boldsymbol{\omega}}\bar A \gt 5$ Hz) yields improved predictability, with $\newcommand{\bialpha}{\boldsymbol{\alpha}}\newcommand{\bibeta}{\boldsymbol{\beta}}\newcommand{\bigamma}{\boldsymbol{\gamma}}\newcommand{\bidelta}{\boldsymbol{\delta}}\newcommand{\bivarepsilon}{\boldsymbol{\varepsilon}}\newcommand{\bizeta}{\boldsymbol{\zeta}}\newcommand{\bieta}{\boldsymbol{\eta}}\newcommand{\bitheta}{\boldsymbol{\theta}}\newcommand{\biiota}{\boldsymbol{\iota}}\newcommand{\bikappa}{\boldsymbol{\kappa}}\newcommand{\bilambda}{\boldsymbol{\lambda}}\newcommand{\bimu}{\boldsymbol{\mu}}\newcommand{\binu}{\boldsymbol{\nu}}\newcommand{\bixi}{\boldsymbol{\xi}}\newcommand{\biomicron}{\boldsymbol{\micron}}\newcommand{\bipi}{\boldsymbol{\pi}}\newcommand{\birho}{\boldsymbol{\rho}}\newcommand{\bisigma}{\boldsymbol{\sigma}}\newcommand{\bitau}{\boldsymbol{\tau}}\newcommand{\biupsilon}{\boldsymbol{\upsilon}}\newcommand{\biphi}{\boldsymbol{\phi}}\newcommand{\bichi}{\boldsymbol{\chi}}\newcommand{\bipsi}{\boldsymbol{\psi}}\newcommand{\biomega}{\boldsymbol{\omega}}\overline {{\rm{CC}}} _{{\rm{norm}}}^{{\rm{CNN2}}} = {0}.{69}\ \pm\ {0}.{01}$, increased for neurons with greater mean firing rates. Similarly, we found that the model performs much better on reliable neurons than on those with low neural reliability. As the limit CC_max_ on predictably set by the neural reliability decreases, the model performance decreases by far more, meaning that overall the model does far worse at predicting the activity of these neurons. Selecting only the reliable neurons, CC_max_ > 0.80, yields improved predictability, with $\newcommand{\bialpha}{\boldsymbol{\alpha}}\newcommand{\bibeta}{\boldsymbol{\beta}}\newcommand{\bigamma}{\boldsymbol{\gamma}}\newcommand{\bidelta}{\boldsymbol{\delta}}\newcommand{\bivarepsilon}{\boldsymbol{\varepsilon}}\newcommand{\bizeta}{\boldsymbol{\zeta}}\newcommand{\bieta}{\boldsymbol{\eta}}\newcommand{\bitheta}{\boldsymbol{\theta}}\newcommand{\biiota}{\boldsymbol{\iota}}\newcommand{\bikappa}{\boldsymbol{\kappa}}\newcommand{\bilambda}{\boldsymbol{\lambda}}\newcommand{\bimu}{\boldsymbol{\mu}}\newcommand{\binu}{\boldsymbol{\nu}}\newcommand{\bixi}{\boldsymbol{\xi}}\newcommand{\biomicron}{\boldsymbol{\micron}}\newcommand{\bipi}{\boldsymbol{\pi}}\newcommand{\birho}{\boldsymbol{\rho}}\newcommand{\bisigma}{\boldsymbol{\sigma}}\newcommand{\bitau}{\boldsymbol{\tau}}\newcommand{\biupsilon}{\boldsymbol{\upsilon}}\newcommand{\biphi}{\boldsymbol{\phi}}\newcommand{\bichi}{\boldsymbol{\chi}}\newcommand{\bipsi}{\boldsymbol{\psi}}\newcommand{\biomega}{\boldsymbol{\omega}}\overline {{\rm{CC}}} _{{\rm{norm}}}^{{\rm{CNN2}}} = {0}.{68}\ \pm\ {0}.{01}$. Thus, we found that our model describes particularly well the neural encoding of both the cells that are more active ($\newcommand{\bialpha}{\boldsymbol{\alpha}}\newcommand{\bibeta}{\boldsymbol{\beta}}\newcommand{\bigamma}{\boldsymbol{\gamma}}\newcommand{\bidelta}{\boldsymbol{\delta}}\newcommand{\bivarepsilon}{\boldsymbol{\varepsilon}}\newcommand{\bizeta}{\boldsymbol{\zeta}}\newcommand{\bieta}{\boldsymbol{\eta}}\newcommand{\bitheta}{\boldsymbol{\theta}}\newcommand{\biiota}{\boldsymbol{\iota}}\newcommand{\bikappa}{\boldsymbol{\kappa}}\newcommand{\bilambda}{\boldsymbol{\lambda}}\newcommand{\bimu}{\boldsymbol{\mu}}\newcommand{\binu}{\boldsymbol{\nu}}\newcommand{\bixi}{\boldsymbol{\xi}}\newcommand{\biomicron}{\boldsymbol{\micron}}\newcommand{\bipi}{\boldsymbol{\pi}}\newcommand{\birho}{\boldsymbol{\rho}}\newcommand{\bisigma}{\boldsymbol{\sigma}}\newcommand{\bitau}{\boldsymbol{\tau}}\newcommand{\biupsilon}{\boldsymbol{\upsilon}}\newcommand{\biphi}{\boldsymbol{\phi}}\newcommand{\bichi}{\boldsymbol{\chi}}\newcommand{\bipsi}{\boldsymbol{\psi}}\newcommand{\biomega}{\boldsymbol{\omega}}\bar A\ \gt\ 5{\rm{Hz}}$) and the neurons that are more reliable (CC_max_ > 0.80).

## Discussion

We trained a deep convolutional neural network to predict the firing rates of neurons in macaque V1 in response to natural image stimuli. In contrast to shallow models, such as linear–nonlinear models that can only describe simple cells, we find that our convolutional neural network can describe a broad range of cells. Firing rates of both orientation-selective and non-orientation-selective neurons can be predicted with high accuracy. Our network describes the more active and more reliable cells particularly well. Additionally, we find that the two-convolutional-layer network outperforms a variety of other models.

Our results take a key step toward cracking the neural code for how visual stimuli are translated into neural activity in V1. This would be a major step forward in sensory neuroscience, and would enable new technologies that could restore sight to the blind. For example, cameras could continuously feed images into networks that would determine the precise V1 activity patterns that correspond to those images: a camera-to-brain translator. Brain-stimulation methods like optogenetics (Ozbay et al., 2015) could then be used to generate those same activity patterns within the brain, thereby restoring sight.

### Model comparisons and depth

Comparing across all of our fully data-driven models (Figure 4B, fully trained) of visual processing in V1, we find that increasing the complexity or depth of the models increases the ability of these models to replicate the visual processes that take place in V1, up to a convolutional neural network with two convolutional layers. Increasing the depth saturates or modestly decreases this CNN2 network's performance. We also find some difference between networks of comparable depths. For instance, the CNN1 and LN-LN networks are both the same depth, with two hidden layers. However, CNN1 does far better at predicting the firing rates in V1. The increased performance of CNN1 is perhaps due to the constraints of the convolutional filters. We want to emphasize that our LN-LN model represents only a small subset of all the possible LN-LN models, and our CNN1 model could be classified as an LN-LN model. Overall, our results support the hypothesis that a model architecture with two convolutional layers and an all-to-all layer well represents the visual processing that takes place in V1.

### Comparisons to other work

Although it is difficult for a variety of reasons to fairly compare the performances of published results, we predict neural activity with performance that is comparable to the state of the art. Over all neurons, the correlation between our network predictions and the actual neural firing rates is $\newcommand{\bialpha}{\boldsymbol{\alpha}}\newcommand{\bibeta}{\boldsymbol{\beta}}\newcommand{\bigamma}{\boldsymbol{\gamma}}\newcommand{\bidelta}{\boldsymbol{\delta}}\newcommand{\bivarepsilon}{\boldsymbol{\varepsilon}}\newcommand{\bizeta}{\boldsymbol{\zeta}}\newcommand{\bieta}{\boldsymbol{\eta}}\newcommand{\bitheta}{\boldsymbol{\theta}}\newcommand{\biiota}{\boldsymbol{\iota}}\newcommand{\bikappa}{\boldsymbol{\kappa}}\newcommand{\bilambda}{\boldsymbol{\lambda}}\newcommand{\bimu}{\boldsymbol{\mu}}\newcommand{\binu}{\boldsymbol{\nu}}\newcommand{\bixi}{\boldsymbol{\xi}}\newcommand{\biomicron}{\boldsymbol{\micron}}\newcommand{\bipi}{\boldsymbol{\pi}}\newcommand{\birho}{\boldsymbol{\rho}}\newcommand{\bisigma}{\boldsymbol{\sigma}}\newcommand{\bitau}{\boldsymbol{\tau}}\newcommand{\biupsilon}{\boldsymbol{\upsilon}}\newcommand{\biphi}{\boldsymbol{\phi}}\newcommand{\bichi}{\boldsymbol{\chi}}\newcommand{\bipsi}{\boldsymbol{\psi}}\newcommand{\biomega}{\boldsymbol{\omega}}\overline {{\rm{CC}}} _{{\rm{abs}}}^{{\rm{CNN2}}} = {0}.{493}\ \pm\ {0}.{014}$. For comparison, Lau, Stanley, and Dan (2002) achieved predictability of $\newcommand{\bialpha}{\boldsymbol{\alpha}}\newcommand{\bibeta}{\boldsymbol{\beta}}\newcommand{\bigamma}{\boldsymbol{\gamma}}\newcommand{\bidelta}{\boldsymbol{\delta}}\newcommand{\bivarepsilon}{\boldsymbol{\varepsilon}}\newcommand{\bizeta}{\boldsymbol{\zeta}}\newcommand{\bieta}{\boldsymbol{\eta}}\newcommand{\bitheta}{\boldsymbol{\theta}}\newcommand{\biiota}{\boldsymbol{\iota}}\newcommand{\bikappa}{\boldsymbol{\kappa}}\newcommand{\bilambda}{\boldsymbol{\lambda}}\newcommand{\bimu}{\boldsymbol{\mu}}\newcommand{\binu}{\boldsymbol{\nu}}\newcommand{\bixi}{\boldsymbol{\xi}}\newcommand{\biomicron}{\boldsymbol{\micron}}\newcommand{\bipi}{\boldsymbol{\pi}}\newcommand{\birho}{\boldsymbol{\rho}}\newcommand{\bisigma}{\boldsymbol{\sigma}}\newcommand{\bitau}{\boldsymbol{\tau}}\newcommand{\biupsilon}{\boldsymbol{\upsilon}}\newcommand{\biphi}{\boldsymbol{\phi}}\newcommand{\bichi}{\boldsymbol{\chi}}\newcommand{\bipsi}{\boldsymbol{\psi}}\newcommand{\biomega}{\boldsymbol{\omega}}{\overline {{\rm{CC}}} _{{\rm{abs}}}} = {0}.{45}$ for simple cells and $\newcommand{\bialpha}{\boldsymbol{\alpha}}\newcommand{\bibeta}{\boldsymbol{\beta}}\newcommand{\bigamma}{\boldsymbol{\gamma}}\newcommand{\bidelta}{\boldsymbol{\delta}}\newcommand{\bivarepsilon}{\boldsymbol{\varepsilon}}\newcommand{\bizeta}{\boldsymbol{\zeta}}\newcommand{\bieta}{\boldsymbol{\eta}}\newcommand{\bitheta}{\boldsymbol{\theta}}\newcommand{\biiota}{\boldsymbol{\iota}}\newcommand{\bikappa}{\boldsymbol{\kappa}}\newcommand{\bilambda}{\boldsymbol{\lambda}}\newcommand{\bimu}{\boldsymbol{\mu}}\newcommand{\binu}{\boldsymbol{\nu}}\newcommand{\bixi}{\boldsymbol{\xi}}\newcommand{\biomicron}{\boldsymbol{\micron}}\newcommand{\bipi}{\boldsymbol{\pi}}\newcommand{\birho}{\boldsymbol{\rho}}\newcommand{\bisigma}{\boldsymbol{\sigma}}\newcommand{\bitau}{\boldsymbol{\tau}}\newcommand{\biupsilon}{\boldsymbol{\upsilon}}\newcommand{\biphi}{\boldsymbol{\phi}}\newcommand{\bichi}{\boldsymbol{\chi}}\newcommand{\bipsi}{\boldsymbol{\psi}}\newcommand{\biomega}{\boldsymbol{\omega}}{\overline {{\rm{CC}}} _{{\rm{abs}}}} = {0}.{31}$ for complex cells; Vintch et al. (2015) achieved predictability of $\newcommand{\bialpha}{\boldsymbol{\alpha}}\newcommand{\bibeta}{\boldsymbol{\beta}}\newcommand{\bigamma}{\boldsymbol{\gamma}}\newcommand{\bidelta}{\boldsymbol{\delta}}\newcommand{\bivarepsilon}{\boldsymbol{\varepsilon}}\newcommand{\bizeta}{\boldsymbol{\zeta}}\newcommand{\bieta}{\boldsymbol{\eta}}\newcommand{\bitheta}{\boldsymbol{\theta}}\newcommand{\biiota}{\boldsymbol{\iota}}\newcommand{\bikappa}{\boldsymbol{\kappa}}\newcommand{\bilambda}{\boldsymbol{\lambda}}\newcommand{\bimu}{\boldsymbol{\mu}}\newcommand{\binu}{\boldsymbol{\nu}}\newcommand{\bixi}{\boldsymbol{\xi}}\newcommand{\biomicron}{\boldsymbol{\micron}}\newcommand{\bipi}{\boldsymbol{\pi}}\newcommand{\birho}{\boldsymbol{\rho}}\newcommand{\bisigma}{\boldsymbol{\sigma}}\newcommand{\bitau}{\boldsymbol{\tau}}\newcommand{\biupsilon}{\boldsymbol{\upsilon}}\newcommand{\biphi}{\boldsymbol{\phi}}\newcommand{\bichi}{\boldsymbol{\chi}}\newcommand{\bipsi}{\boldsymbol{\psi}}\newcommand{\biomega}{\boldsymbol{\omega}}{\overline {{\rm{CC}}} _{{\rm{abs}}}} = {0}.{55}$ for simple cells and $\newcommand{\bialpha}{\boldsymbol{\alpha}}\newcommand{\bibeta}{\boldsymbol{\beta}}\newcommand{\bigamma}{\boldsymbol{\gamma}}\newcommand{\bidelta}{\boldsymbol{\delta}}\newcommand{\bivarepsilon}{\boldsymbol{\varepsilon}}\newcommand{\bizeta}{\boldsymbol{\zeta}}\newcommand{\bieta}{\boldsymbol{\eta}}\newcommand{\bitheta}{\boldsymbol{\theta}}\newcommand{\biiota}{\boldsymbol{\iota}}\newcommand{\bikappa}{\boldsymbol{\kappa}}\newcommand{\bilambda}{\boldsymbol{\lambda}}\newcommand{\bimu}{\boldsymbol{\mu}}\newcommand{\binu}{\boldsymbol{\nu}}\newcommand{\bixi}{\boldsymbol{\xi}}\newcommand{\biomicron}{\boldsymbol{\micron}}\newcommand{\bipi}{\boldsymbol{\pi}}\newcommand{\birho}{\boldsymbol{\rho}}\newcommand{\bisigma}{\boldsymbol{\sigma}}\newcommand{\bitau}{\boldsymbol{\tau}}\newcommand{\biupsilon}{\boldsymbol{\upsilon}}\newcommand{\biphi}{\boldsymbol{\phi}}\newcommand{\bichi}{\boldsymbol{\chi}}\newcommand{\bipsi}{\boldsymbol{\psi}}\newcommand{\biomega}{\boldsymbol{\omega}}{\overline {{\rm{CC}}} _{{\rm{abs}}}} = 0.42$ for complex cells; and Prenger, Wu, David, and Gallant (2004) achieved $\newcommand{\bialpha}{\boldsymbol{\alpha}}\newcommand{\bibeta}{\boldsymbol{\beta}}\newcommand{\bigamma}{\boldsymbol{\gamma}}\newcommand{\bidelta}{\boldsymbol{\delta}}\newcommand{\bivarepsilon}{\boldsymbol{\varepsilon}}\newcommand{\bizeta}{\boldsymbol{\zeta}}\newcommand{\bieta}{\boldsymbol{\eta}}\newcommand{\bitheta}{\boldsymbol{\theta}}\newcommand{\biiota}{\boldsymbol{\iota}}\newcommand{\bikappa}{\boldsymbol{\kappa}}\newcommand{\bilambda}{\boldsymbol{\lambda}}\newcommand{\bimu}{\boldsymbol{\mu}}\newcommand{\binu}{\boldsymbol{\nu}}\newcommand{\bixi}{\boldsymbol{\xi}}\newcommand{\biomicron}{\boldsymbol{\micron}}\newcommand{\bipi}{\boldsymbol{\pi}}\newcommand{\birho}{\boldsymbol{\rho}}\newcommand{\bisigma}{\boldsymbol{\sigma}}\newcommand{\bitau}{\boldsymbol{\tau}}\newcommand{\biupsilon}{\boldsymbol{\upsilon}}\newcommand{\biphi}{\boldsymbol{\phi}}\newcommand{\bichi}{\boldsymbol{\chi}}\newcommand{\bipsi}{\boldsymbol{\psi}}\newcommand{\biomega}{\boldsymbol{\omega}}{\overline {{\rm{CC}}} _{{\rm{abs}}}} = {0}.{24}$ averaged over all cells. Lehky et al. (1992) achieved $\newcommand{\bialpha}{\boldsymbol{\alpha}}\newcommand{\bibeta}{\boldsymbol{\beta}}\newcommand{\bigamma}{\boldsymbol{\gamma}}\newcommand{\bidelta}{\boldsymbol{\delta}}\newcommand{\bivarepsilon}{\boldsymbol{\varepsilon}}\newcommand{\bizeta}{\boldsymbol{\zeta}}\newcommand{\bieta}{\boldsymbol{\eta}}\newcommand{\bitheta}{\boldsymbol{\theta}}\newcommand{\biiota}{\boldsymbol{\iota}}\newcommand{\bikappa}{\boldsymbol{\kappa}}\newcommand{\bilambda}{\boldsymbol{\lambda}}\newcommand{\bimu}{\boldsymbol{\mu}}\newcommand{\binu}{\boldsymbol{\nu}}\newcommand{\bixi}{\boldsymbol{\xi}}\newcommand{\biomicron}{\boldsymbol{\micron}}\newcommand{\bipi}{\boldsymbol{\pi}}\newcommand{\birho}{\boldsymbol{\rho}}\newcommand{\bisigma}{\boldsymbol{\sigma}}\newcommand{\bitau}{\boldsymbol{\tau}}\newcommand{\biupsilon}{\boldsymbol{\upsilon}}\newcommand{\biphi}{\boldsymbol{\phi}}\newcommand{\bichi}{\boldsymbol{\chi}}\newcommand{\bipsi}{\boldsymbol{\psi}}\newcommand{\biomega}{\boldsymbol{\omega}}{\overline {{\rm{CC}}} _{{\rm{abs}}}} = {0}.{78}$, and Willmore, Prenger, and Gallant (2010) achieved a predictability (quantified as fraction of variance explained) of 0.4. However, some contextual factors confound direct comparison to these results. Specifically, Lehky et al. selected neurons that are easier to predict by specifically choosing neurons that responded strongly to the presentation of bars of light; Vintch et al. analyzed direction-selective neurons; and Willmore et al. adjusted their image to match the receptive field of each neuron they predicted. We, by contrast, neither tailored our stimulation to our neurons nor selected well-behaved neurons. By selecting on either reliability or activation, we could easily achieve $\newcommand{\bialpha}{\boldsymbol{\alpha}}\newcommand{\bibeta}{\boldsymbol{\beta}}\newcommand{\bigamma}{\boldsymbol{\gamma}}\newcommand{\bidelta}{\boldsymbol{\delta}}\newcommand{\bivarepsilon}{\boldsymbol{\varepsilon}}\newcommand{\bizeta}{\boldsymbol{\zeta}}\newcommand{\bieta}{\boldsymbol{\eta}}\newcommand{\bitheta}{\boldsymbol{\theta}}\newcommand{\biiota}{\boldsymbol{\iota}}\newcommand{\bikappa}{\boldsymbol{\kappa}}\newcommand{\bilambda}{\boldsymbol{\lambda}}\newcommand{\bimu}{\boldsymbol{\mu}}\newcommand{\binu}{\boldsymbol{\nu}}\newcommand{\bixi}{\boldsymbol{\xi}}\newcommand{\biomicron}{\boldsymbol{\micron}}\newcommand{\bipi}{\boldsymbol{\pi}}\newcommand{\birho}{\boldsymbol{\rho}}\newcommand{\bisigma}{\boldsymbol{\sigma}}\newcommand{\bitau}{\boldsymbol{\tau}}\newcommand{\biupsilon}{\boldsymbol{\upsilon}}\newcommand{\biphi}{\boldsymbol{\phi}}\newcommand{\bichi}{\boldsymbol{\chi}}\newcommand{\bipsi}{\boldsymbol{\psi}}\newcommand{\biomega}{\boldsymbol{\omega}}\overline {{\rm{CC}}} _{{\rm{abs}}}^{{\rm{CNN2}}}\ \gt\ {0}.{6}$.

Consistent with Cadena et al. (2017), we find that the VGG layer most predictive of V1 neural firing rates is Conv3,1. However, in contrast with Cadena et al., we find that our data-driven CNN outperforms even this best VGG layer. In this comparison, confounds include having different images sets, using different methods for optimizing hyperparameters of CNNs, and using anesthetized monkeys rather than awake monkeys.

### Identifying visual features that cause the neurons to spike

In addition to making predictions of neural activity, the CNN represents the underlying visual processing that drives the population of neurons to spike. As an example of how to use the model as a tool to investigate the functions of individual neurons, we used DeepDream-like techniques (Mahendran & Vedaldi, 2015) to identify the visual features that cause each cell to spike. We inverted our network by finding input images that cause a given cell to spike at a prespecified level. To do this, we first took the fully trained network and set Gaussian-white-noise images as the input. We then used back-propagation to modify the pixel values of the input image to push the chosen neuron's predicted firing rate toward the prespecified level. Thus, we found an input image that induced the prespecified response.

**Figure 6:**
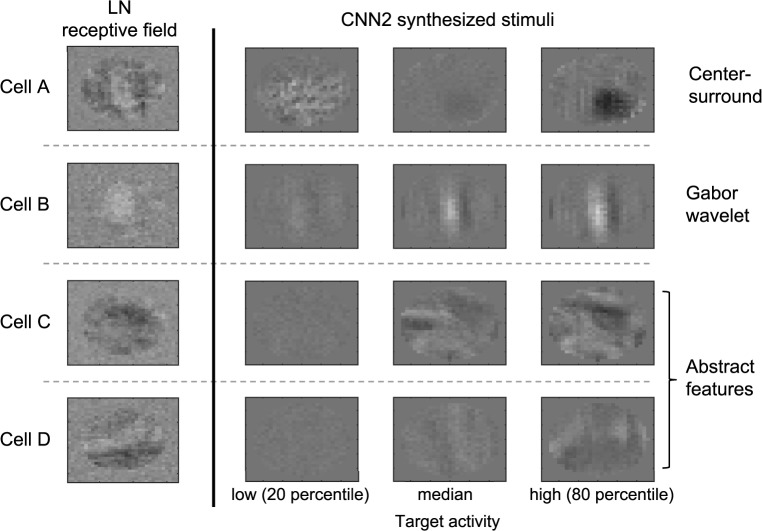
Using the network model to reveal the visual features that drive individual neurons. (Left) Receptive-field filters from the LN model for four neurons. (Right) For each neuron, we synthesized images that drove the predicted firing rates to the specified target values using the convolutional-neural-network model. These target firing rates were chosen to be different percentiles of the neuron's firing-rate distribution. Cells A and B appear to respond to localized image features, whereas cells C and D respond to more abstract image features.

We applied this procedure to several different neurons that are well described by the model, and at several different target firing rates (Figure 6). Cells A ($\newcommand{\bialpha}{\boldsymbol{\alpha}}\newcommand{\bibeta}{\boldsymbol{\beta}}\newcommand{\bigamma}{\boldsymbol{\gamma}}\newcommand{\bidelta}{\boldsymbol{\delta}}\newcommand{\bivarepsilon}{\boldsymbol{\varepsilon}}\newcommand{\bizeta}{\boldsymbol{\zeta}}\newcommand{\bieta}{\boldsymbol{\eta}}\newcommand{\bitheta}{\boldsymbol{\theta}}\newcommand{\biiota}{\boldsymbol{\iota}}\newcommand{\bikappa}{\boldsymbol{\kappa}}\newcommand{\bilambda}{\boldsymbol{\lambda}}\newcommand{\bimu}{\boldsymbol{\mu}}\newcommand{\binu}{\boldsymbol{\nu}}\newcommand{\bixi}{\boldsymbol{\xi}}\newcommand{\biomicron}{\boldsymbol{\micron}}\newcommand{\bipi}{\boldsymbol{\pi}}\newcommand{\birho}{\boldsymbol{\rho}}\newcommand{\bisigma}{\boldsymbol{\sigma}}\newcommand{\bitau}{\boldsymbol{\tau}}\newcommand{\biupsilon}{\boldsymbol{\upsilon}}\newcommand{\biphi}{\boldsymbol{\phi}}\newcommand{\bichi}{\boldsymbol{\chi}}\newcommand{\bipsi}{\boldsymbol{\psi}}\newcommand{\biomega}{\boldsymbol{\omega}}{\rm{CC}}_{{\rm{abs}}}^{{\rm{CNN2}}} = {0}.{88}$) and B ($\newcommand{\bialpha}{\boldsymbol{\alpha}}\newcommand{\bibeta}{\boldsymbol{\beta}}\newcommand{\bigamma}{\boldsymbol{\gamma}}\newcommand{\bidelta}{\boldsymbol{\delta}}\newcommand{\bivarepsilon}{\boldsymbol{\varepsilon}}\newcommand{\bizeta}{\boldsymbol{\zeta}}\newcommand{\bieta}{\boldsymbol{\eta}}\newcommand{\bitheta}{\boldsymbol{\theta}}\newcommand{\biiota}{\boldsymbol{\iota}}\newcommand{\bikappa}{\boldsymbol{\kappa}}\newcommand{\bilambda}{\boldsymbol{\lambda}}\newcommand{\bimu}{\boldsymbol{\mu}}\newcommand{\binu}{\boldsymbol{\nu}}\newcommand{\bixi}{\boldsymbol{\xi}}\newcommand{\biomicron}{\boldsymbol{\micron}}\newcommand{\bipi}{\boldsymbol{\pi}}\newcommand{\birho}{\boldsymbol{\rho}}\newcommand{\bisigma}{\boldsymbol{\sigma}}\newcommand{\bitau}{\boldsymbol{\tau}}\newcommand{\biupsilon}{\boldsymbol{\upsilon}}\newcommand{\biphi}{\boldsymbol{\phi}}\newcommand{\bichi}{\boldsymbol{\chi}}\newcommand{\bipsi}{\boldsymbol{\psi}}\newcommand{\biomega}{\boldsymbol{\omega}}{\rm{CC}}_{{\rm{abs}}}^{{\rm{CNN2}}} = {0}.{89}$) appear to function like previously characterized cells. Cell A responds to a center–surround image feature, and cell B's receptive field is a Gabor wavelet. In contrast, cells C ($\newcommand{\bialpha}{\boldsymbol{\alpha}}\newcommand{\bibeta}{\boldsymbol{\beta}}\newcommand{\bigamma}{\boldsymbol{\gamma}}\newcommand{\bidelta}{\boldsymbol{\delta}}\newcommand{\bivarepsilon}{\boldsymbol{\varepsilon}}\newcommand{\bizeta}{\boldsymbol{\zeta}}\newcommand{\bieta}{\boldsymbol{\eta}}\newcommand{\bitheta}{\boldsymbol{\theta}}\newcommand{\biiota}{\boldsymbol{\iota}}\newcommand{\bikappa}{\boldsymbol{\kappa}}\newcommand{\bilambda}{\boldsymbol{\lambda}}\newcommand{\bimu}{\boldsymbol{\mu}}\newcommand{\binu}{\boldsymbol{\nu}}\newcommand{\bixi}{\boldsymbol{\xi}}\newcommand{\biomicron}{\boldsymbol{\micron}}\newcommand{\bipi}{\boldsymbol{\pi}}\newcommand{\birho}{\boldsymbol{\rho}}\newcommand{\bisigma}{\boldsymbol{\sigma}}\newcommand{\bitau}{\boldsymbol{\tau}}\newcommand{\biupsilon}{\boldsymbol{\upsilon}}\newcommand{\biphi}{\boldsymbol{\phi}}\newcommand{\bichi}{\boldsymbol{\chi}}\newcommand{\bipsi}{\boldsymbol{\psi}}\newcommand{\biomega}{\boldsymbol{\omega}}{\rm{CC}}_{{\rm{abs}}}^{{\rm{CNN2}}} = {0}.{91}$) and D ($\newcommand{\bialpha}{\boldsymbol{\alpha}}\newcommand{\bibeta}{\boldsymbol{\beta}}\newcommand{\bigamma}{\boldsymbol{\gamma}}\newcommand{\bidelta}{\boldsymbol{\delta}}\newcommand{\bivarepsilon}{\boldsymbol{\varepsilon}}\newcommand{\bizeta}{\boldsymbol{\zeta}}\newcommand{\bieta}{\boldsymbol{\eta}}\newcommand{\bitheta}{\boldsymbol{\theta}}\newcommand{\biiota}{\boldsymbol{\iota}}\newcommand{\bikappa}{\boldsymbol{\kappa}}\newcommand{\bilambda}{\boldsymbol{\lambda}}\newcommand{\bimu}{\boldsymbol{\mu}}\newcommand{\binu}{\boldsymbol{\nu}}\newcommand{\bixi}{\boldsymbol{\xi}}\newcommand{\biomicron}{\boldsymbol{\micron}}\newcommand{\bipi}{\boldsymbol{\pi}}\newcommand{\birho}{\boldsymbol{\rho}}\newcommand{\bisigma}{\boldsymbol{\sigma}}\newcommand{\bitau}{\boldsymbol{\tau}}\newcommand{\biupsilon}{\boldsymbol{\upsilon}}\newcommand{\biphi}{\boldsymbol{\phi}}\newcommand{\bichi}{\boldsymbol{\chi}}\newcommand{\bipsi}{\boldsymbol{\psi}}\newcommand{\biomega}{\boldsymbol{\omega}}{\rm{CC}}_{{\rm{abs}}}^{{\rm{CNN2}}} = {0}.{90}$) appear to respond to more abstract image features that are not well represented by simple localized image masks. For comparison, we plot the receptive fields according to the LN model (Figure 6, left).

By inverting our network, we showed that we can use it as a tool to investigate neurons' response properties that cannot be found with shallower models. Going forward, this method shows potential for characterizing the response properties of more cells in V1 and precisely defining functional cell types that have been previously overlooked. Looking beyond V1, these methods could be applied to understanding higher level cortical processing, such as visual encoding in V2. By finding the features that elicit a response in V2 neurons, this tool could help fill the visual-encoding knowledge gap (Ziemba, Freeman, Movshon, & Simoncelli, 2016) that exists between the abstract encoding of inferior temporal cortex and V4 and the low-level encoding of the retina and V1.

### Window length for firing-rate estimate and most well-isolated neurons

In our main analysis, we focused on predicting the initial neural response to exclude influence of top-down feedback from higher cortical areas. That is, we focused on the timescale when biological neural processing is most analogous to the feed-forward architecture of the artificial neural networks in our study. Because we considered only the initial response of the neurons to the stimulus, we were motivated to ask how well our network architecture can predict the neurons' firing rates, estimated by counting spikes over the full 100-ms window in the data of Coen-Cagli et al. (2015). Repeating our analysis with 100-ms windowed data, we found that our predictions have correlation $\newcommand{\bialpha}{\boldsymbol{\alpha}}\newcommand{\bibeta}{\boldsymbol{\beta}}\newcommand{\bigamma}{\boldsymbol{\gamma}}\newcommand{\bidelta}{\boldsymbol{\delta}}\newcommand{\bivarepsilon}{\boldsymbol{\varepsilon}}\newcommand{\bizeta}{\boldsymbol{\zeta}}\newcommand{\bieta}{\boldsymbol{\eta}}\newcommand{\bitheta}{\boldsymbol{\theta}}\newcommand{\biiota}{\boldsymbol{\iota}}\newcommand{\bikappa}{\boldsymbol{\kappa}}\newcommand{\bilambda}{\boldsymbol{\lambda}}\newcommand{\bimu}{\boldsymbol{\mu}}\newcommand{\binu}{\boldsymbol{\nu}}\newcommand{\bixi}{\boldsymbol{\xi}}\newcommand{\biomicron}{\boldsymbol{\micron}}\newcommand{\bipi}{\boldsymbol{\pi}}\newcommand{\birho}{\boldsymbol{\rho}}\newcommand{\bisigma}{\boldsymbol{\sigma}}\newcommand{\bitau}{\boldsymbol{\tau}}\newcommand{\biupsilon}{\boldsymbol{\upsilon}}\newcommand{\biphi}{\boldsymbol{\phi}}\newcommand{\bichi}{\boldsymbol{\chi}}\newcommand{\bipsi}{\boldsymbol{\psi}}\newcommand{\biomega}{\boldsymbol{\omega}}\overline {{\rm{CC}}} _{{\rm{norm}}}^{{\rm{CNN2}}} = {0}.{506}\ \pm\ {0}.{006}$ to the measured firing rate over all neurons. This is slightly worse than our main analysis, where we used a 50-ms window. This result is not surprising, because we optimized the hyperparameters of our model using a 50-ms window.

Because the data set we use groups both well-isolated neurons and small multiunit clusters, we were motivated to see how our best CNN2 model performs at predicting firing rates of each of these unit types. Following Coen-Cagli et al. (2015), we identified the most well-isolated neurons by choosing only those whose signal-to-noise ratio in the spike sorting is greater than 2.75, and the remaining neurons (spike-sorting signal-to-noise ratio < 2.75) are an indistinguishable mixture of small multiunit clusters and single neurons. We found that the most well-isolated neurons have a predictor performance of $\newcommand{\bialpha}{\boldsymbol{\alpha}}\newcommand{\bibeta}{\boldsymbol{\beta}}\newcommand{\bigamma}{\boldsymbol{\gamma}}\newcommand{\bidelta}{\boldsymbol{\delta}}\newcommand{\bivarepsilon}{\boldsymbol{\varepsilon}}\newcommand{\bizeta}{\boldsymbol{\zeta}}\newcommand{\bieta}{\boldsymbol{\eta}}\newcommand{\bitheta}{\boldsymbol{\theta}}\newcommand{\biiota}{\boldsymbol{\iota}}\newcommand{\bikappa}{\boldsymbol{\kappa}}\newcommand{\bilambda}{\boldsymbol{\lambda}}\newcommand{\bimu}{\boldsymbol{\mu}}\newcommand{\binu}{\boldsymbol{\nu}}\newcommand{\bixi}{\boldsymbol{\xi}}\newcommand{\biomicron}{\boldsymbol{\micron}}\newcommand{\bipi}{\boldsymbol{\pi}}\newcommand{\birho}{\boldsymbol{\rho}}\newcommand{\bisigma}{\boldsymbol{\sigma}}\newcommand{\bitau}{\boldsymbol{\tau}}\newcommand{\biupsilon}{\boldsymbol{\upsilon}}\newcommand{\biphi}{\boldsymbol{\phi}}\newcommand{\bichi}{\boldsymbol{\chi}}\newcommand{\bipsi}{\boldsymbol{\psi}}\newcommand{\biomega}{\boldsymbol{\omega}}\overline {{\rm{CC}}} _{{\rm{norm}}}^{{\rm{CNN2}}} = {0}.{414}\ \pm\ {0}.{016}$, whereas the mixture of clusters and single neurons has $\newcommand{\bialpha}{\boldsymbol{\alpha}}\newcommand{\bibeta}{\boldsymbol{\beta}}\newcommand{\bigamma}{\boldsymbol{\gamma}}\newcommand{\bidelta}{\boldsymbol{\delta}}\newcommand{\bivarepsilon}{\boldsymbol{\varepsilon}}\newcommand{\bizeta}{\boldsymbol{\zeta}}\newcommand{\bieta}{\boldsymbol{\eta}}\newcommand{\bitheta}{\boldsymbol{\theta}}\newcommand{\biiota}{\boldsymbol{\iota}}\newcommand{\bikappa}{\boldsymbol{\kappa}}\newcommand{\bilambda}{\boldsymbol{\lambda}}\newcommand{\bimu}{\boldsymbol{\mu}}\newcommand{\binu}{\boldsymbol{\nu}}\newcommand{\bixi}{\boldsymbol{\xi}}\newcommand{\biomicron}{\boldsymbol{\micron}}\newcommand{\bipi}{\boldsymbol{\pi}}\newcommand{\birho}{\boldsymbol{\rho}}\newcommand{\bisigma}{\boldsymbol{\sigma}}\newcommand{\bitau}{\boldsymbol{\tau}}\newcommand{\biupsilon}{\boldsymbol{\upsilon}}\newcommand{\biphi}{\boldsymbol{\phi}}\newcommand{\bichi}{\boldsymbol{\chi}}\newcommand{\bipsi}{\boldsymbol{\psi}}\newcommand{\biomega}{\boldsymbol{\omega}}\overline {{\rm{CC}}} _{{\rm{norm}}}^{{\rm{CNN2}}} = {0}.{635}\ \pm\ {0}.{012}$. We were initially surprised by this finding, as we expected the well-isolated single units to be the most predictable. However, the multiunit clusters, being aggregates of several neurons, have higher average firing rates: 12.6 ± 0.6 spikes/s on average (*M* ± *SEM*), compared with 8.4 ± 0.8 spikes/s for the well-isolated single units (estimated during the 50-ms spike-counting window). Recall that neurons with higher firing rates were generally more predictable (Figure 5C). We thus attribute the higher predictability of the multiunit clusters to their higher mean firing rates.

## Footnotes

1 Our model is publicly available at https://github.com/jzlab/v1_predictor.

2 In our experience, SAILnet is much faster to train than SparseNet. We used the publicly available SAILnet code out of the box (http://www.jzlab.org/sailcodes.html), without changing any parameter values except image size.
